# Potential limitations of microdystrophin gene therapy for Duchenne muscular dystrophy

**DOI:** 10.1172/jci.insight.165869

**Published:** 2024-05-07

**Authors:** Cora C. Hart, Young il Lee, Jun Xie, Guangping Gao, Brian L. Lin, David W. Hammers, H. Lee Sweeney

**Affiliations:** 1 Department of Pharmacology & Therapeutics and; 2 Myology Institute, University of Florida College of Medicine, Gainesville, Florida, USA.; 3 Horae Gene Therapy Center, University of Massachusetts Medical School, Worchester, Massachusetts, USA.; 4 Department of Cell Biology, Neurobiology, and Anatomy & Department of Pediatrics, Medical College of Wisconsin, Milwaukee, Wisconsin, USA.

**Keywords:** Muscle biology, Therapeutics, Gene therapy

## Abstract

Clinical trials delivering high doses of adeno-associated viruses (AAVs) expressing truncated dystrophin molecules (microdystrophins) are underway for Duchenne muscular dystrophy (DMD). We examined the efficiency and efficacy of this strategy with 4 microdystrophin constructs (3 in clinical trials and a variant of the largest clinical construct), in a severe mouse model of DMD, using AAV doses comparable with those in clinical trials. We achieved high levels of microdystrophin expression in striated muscles with cardiac expression approximately 10-fold higher than that observed in skeletal muscle. Significant, albeit incomplete, correction of skeletal muscle disease was observed. Surprisingly, a lethal acceleration of cardiac disease occurred with 2 of the microdystrophins. The detrimental cardiac effect appears to be caused by variable competition (dependent on microdystrophin design and expression level) between microdystrophin and utrophin at the cardiomyocyte membrane. There may also be a contribution from an overloading of protein degradation. The significance of these observations for patients currently being treated with AAV-microdystrophin therapies is unclear since the levels of expression being achieved in the DMD hearts are unknown. However, these findings suggest that microdystrophin treatments need to avoid excessively high levels of expression in the heart and that cardiac function should be carefully monitored in these patients.

## Abstract 2

Long-tern evaluation of the three AAV-microdystrophin gene therapy constructs currently in US clinical trials reveals skeletal and cardiac muscle pathology in a murine Duchenne muscular dystrophy model.

## Introduction

Duchenne muscular dystrophy (DMD) is an X-linked disorder that affects approximately 1 in 5,000 newborn males (1). It is the most common of the childhood muscular dystrophies and results from the lack of the membrane-associated protein, dystrophin, which is critical for proper force transmission in muscle cells (2, 3). The loss of dystrophin results in hypersensitivity to injury in the skeletal muscle and leads to cardiac dysfunction. The skeletal muscle initially undergoes rounds of injury and repair, but repair eventually begins to fail, and the muscles are replaced with fibrosis and fat. The muscle loss progresses from proximal to distal, with the loss of respiratory muscles and/or heart failure as the cause of death, generally in the second or third decade of life (4). The cardiac disease manifests first with diastolic dysfunction and later progresses to a dilated cardiomyopathy (DCM) and failure (5–8).

Gene therapy for DMD has entered the clinic in the form of several versions of a highly truncated dystrophin (microdystrophin) delivered via adeno-associated virus (AAV). While AAV is highly efficient at infecting and transducing striated muscle, its small packaging capacity (~5 kb) makes it impossible to accommodate the full-length dystrophin coding sequence (~14 kb). This has necessitated using AAV to deliver the coding sequence of a highly truncated dystrophin (9, 10) or using AAV to alter splicing of an out-of-frame dystrophin mRNA to create a deletion that restores the proper reading frame (11, 12). In either case, the goal is to express a truncated version of dystrophin to slow disease progression. This strategy essentially aims to transform DMD into a slower-progressing muscular dystrophy, potentially more like some forms of Becker muscular dystrophy (BMD), a disease caused by dystrophin mutations that create in-frame transcripts resulting in production of a variety of truncated forms of dystrophin that are associated with different rates of disease progression.

A number of questions surround the outcome of these trials, particularly the dosing and the potential efficacy of each of the different microdystrophin constructs currently in trial. It is unclear when or if there will be a need to redeliver the therapy due either to dilution of transduced nuclei from muscle growth or due to skeletal muscle turnover because of residual muscle degeneration or general myonuclear loss, resulting in eventual loss of the AAV DNA encoding the microdystrophin transgene. Thus, a major clinical goal is to express the microdystrophin at high levels throughout the skeletal and cardiac muscles; this expression will potentially limit the frequency of needing to redeliver AAV to the skeletal muscle. Since the cardiomyocytes do not turnover, redelivery will be unnecessary unless they were not adequately transduced with the first dose of virus.

Most of the preclinical work supporting these DMD trials was performed using dystrophic mice of C57-based genetic backgrounds, which exhibit mild disease progression when compared with that of other mouse genetic backgrounds (13) and larger mammals (14, 15). While information concerning transgene delivery and expression can be gathered using these C57-based models, it is difficult to assess the translational efficacy of AAV-microdystrophin gene therapies at correcting a severe, life-limiting striated muscle disease. Indeed, the lack of an animal model that is completely representative of the human disease has contributed to the discrepancy in results between preclinical and clinical research and has ultimately resulted in the termination of several DMD clinical trials (16). Therefore, this study utilized a severe mouse model of DMD, the D2.*mdx* mouse harboring the *mdx* mutation on the DBA/2J genetic background (13, 17, 18), to evaluate the long-term effect of AAV-driven microdystrophin on the heart and skeletal muscles in the face of a more aggressive disease progression.

**Figure 1:**
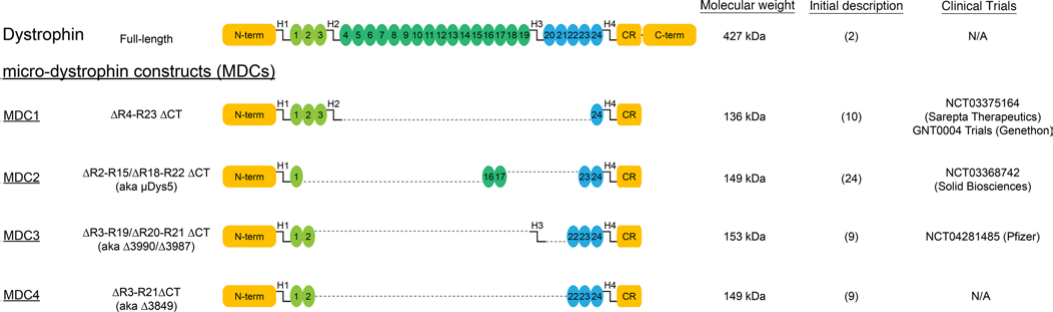
Structure of dystrophin and microdystrophin constructs. A schematic diagram of full-length dystrophin, the microdystrophin constructs (MDCs) currently utilized in clinical trials (MDC1–3), as well as of MDC4, a modification of MDC3.

Common features of microdystrophin constructs (Figure 1) include the N-terminal actin-binding region, 4–5 of the 24 spectrin-like triple helical bundles that make up the rod region, and a truncated C-terminus containing the β-dystroglycan binding site. In this work, we sought to directly compare the efficiency and long-term efficacy of 3 clinical versions, which we refer to as MDC1, MDC2, and MDC3 (Figure 1). Given the size of the promoter (CK8) we used for these comparisons, the size of MDC3 exceeded the efficient packaging limit of AAV. Thus, we also included a smaller, published variant of MDC3 that differs only by the deletion of hinge (H) 3 (MDC4; aka Δ3849; ref. 9). This smaller variant showed no significant difference in efficacy compared with MDC3 in the initial report (9) and demonstrated skeletal muscle rescue in a C57-based transgenic model (19).

**Figure 2:**
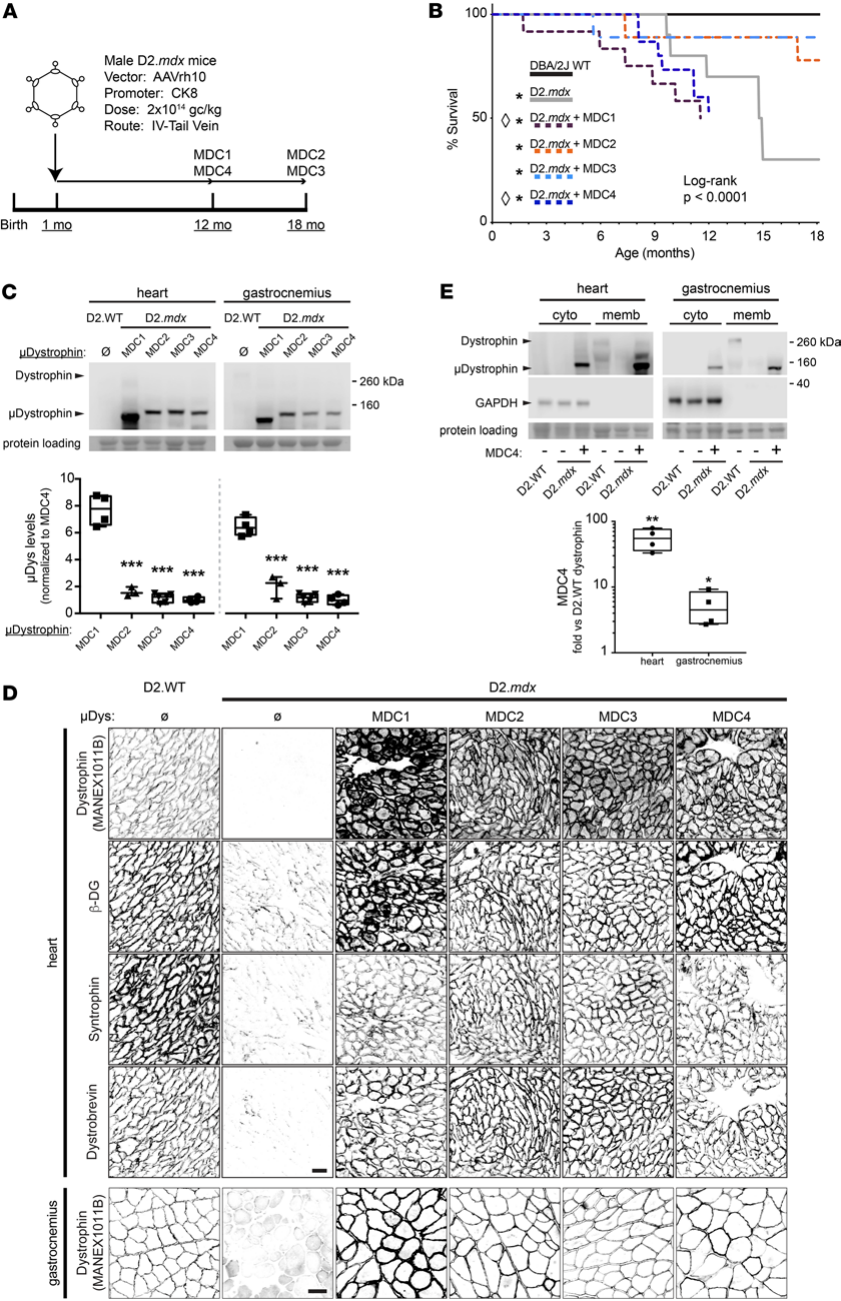
Adeno-associated virus-mediated striated muscle expression of micro-dystrophin constructs. (**A**) Male D2.mdx mice were injected via tail vein with AAV carrying 1 of 4 CK8-driven MCDs (2x1014 gc/kg) at 1 month of age. (**B**) Microdystrophin gene therapy can result in premature death. Survival curve of D2.WT and D2.mdx untreated or treated with each of the 4 MDCs. None of the MDCs restored the lifespan of treated animals to that observed with DBA/2J WT animals (pair-wise Log-rank test; *P < 0.05 vs DBA/2J WT; Bonferroni correction). Two microdystrophin constructs (MDC1 and MDC4) led to premature death of treated mice (pair-wise Log-rank test; ^◊^*P* < 0.05 vs D2.mdx; Bonferroni correction). (**C**) Western blots of lysates of heart and gastrocnemius muscles from D2.mdx animals each transduced with 1 of the 4 MDCs. The 5-repeat MDCs examined (MDC2-4) show similar expression in both muscles, while the 4-repeat MDC1 was expressed at levels several-fold higher in comparison (~8- and ~6-fold higher in heart and gastrocnemius, respectively; *n* = 3–6, *P* < 0.001, 1-way ANOVA; ***P < 0.001 vs. mDC1, Tukey post hoc comparison). (**D**) Top 4 rows: Antibody-mediated labeling of heart transverse sections from D2.WT, D2.mdx untreated or treated with each of the 4 MDCs revealed sarcolemmal localization of microdystrophin proteins and restores sarcolemmal DGC. Bottom row: Antibody-mediated dystrophin labeling of gastrocnemius transverse sections demonstrated maintained sarcolemmal localization of each of the 4 MDCs that mirror sarcolemmal localization of endogenous dystrophin protein until the study endpoints. (**E**) Comparison of MDC4 expression vs endogenous full-length dystrophin in heart and gastrocnemius showed ~50- and ~5-fold overexpression, respectively (*n* = 4, Student’s *t* test, *P < 0.05, **P < 0.01). A majority of MDC4 was found to be associated with the membrane-enriched fraction of each tissue. Box-and-Whisker plots: minimum-to-maximum with 2nd and 3rd quartiles within the box, with a line that indicates the mean. Scale bars: 100 µm.

In this head-to-head evaluation, we sought to determine the long-term efficacy of these 4 microdystrophins at correcting the skeletal and cardiac muscle pathologies associated with the D2.*mdx* mouse model of DMD. As depicted in Figure 2A, this experiment consisted of male D2.*mdx* mice receiving an i.v. delivered dose of AAV-packaged, codon-optimized human microdystrophin at 1 month of age. All constructs were placed behind the CK8 striated muscle promoter (20) and packaged in AAVrh10 serotype vector, which has a high tropism for striated muscle (21) and shares 98.8% of its identity with AAVrh74, a vector utilized in 1 microdystrophin clinical trial (22).

Using a clinical AAV dose (2 × 10^14^ gc/kg; ref. 23), we observed widespread transduction and sustained expression of all 4 microdystrophins in skeletal and cardiac muscles of D2.*mdx* mice with the heart achieving much greater overexpression —compared with endogenous dystrophin — that is ~5- to 10-fold higher than in skeletal muscles. All treatments slowed skeletal muscle disease progression to some degree, although they did not completely stop it. Surprisingly, the overexpression of 2 of the microdystrophins (MDC1 and MDC4; Figure 1) led to an accelerated onset of a DCM, heart failure, and death. These mouse studies highlight the differential long-term efficacy achieved by different microdystrophin constructs but also highlight caution against their overexpression in the heart. As we demonstrate, achieving high-level expression of microdystrophin in the heart may be deleterious, depending on the construct design.

## Results

### Clinical AAV doses enable widespread expression in D2.mdx striated muscle.

As depicted in the experimental schematic in Figure 2A, AAV was administered systemically through the tail vein at a dose of 2 × 10^14^ gc/kg, which is currently used in the clinic (23). The treatment of D2.*mdx* mice in this manner resulted in equivalent striated muscle expression of the 3 largest microdystrophins, MDC2, MDC3, and MDC4 (Figure 2C). The smallest construct, MDC1, achieved much higher levels of expression in striated muscle (~7-fold greater; Figure 2C). We observed robust and uniform expression of all microdystrophins at the sarcolemma of cardiomyocytes, as detected by immunofluorescence (Figure 2D, top panel). The expression of microdystrophin coincided with an increase in membrane-associated content of the dystrophin-glycoprotein complex (DGC) members β-dystroglycan, syntrophin, and dystrobrevin (Figure 2D).

Immunoblotting data estimate that the microdystrophin levels achieved by this treatment for the 3 largest microdystrophins greatly exceed WT levels of native dystrophin in both the gastrocnemius and heart (~5- and ~55-fold greater, respectively; Figure 2E). The relatively high expression level of MDC1 in comparison with the other microdystrophins is not due to high viral transduction, since vector genome content in the heart is not proportional to protein levels (Supplemental Figure 1; supplemental material available online with this article; https://doi.org/10.1172/jci.insight.165869DS1). These results demonstrate that the treatment of D2.*mdx* mice with clinical doses of AAV-packaged microdystrophin leads to efficient transduction and microdystrophin expression in both skeletal and cardiac muscle. Despite equivalent microdystrophin levels being achieved by the 3 largest constructs, we observed a striking difference in survival age between the treatment groups; MDCs 1 and 4 lead to a premature death (Figure 2B). Therefore, the terminal measures for surviving mice receiving MDC1 and MDC4 were conducted at 12 months, while those for MDC2 and MDC3 treatments occurred at 18 months of age, with appropriate age-matched controls for each endpoint (Figure 2A).

### Microdystrophin gene therapy partially corrects the D2.mdx skeletal muscle pathology.

**Figure 3:**
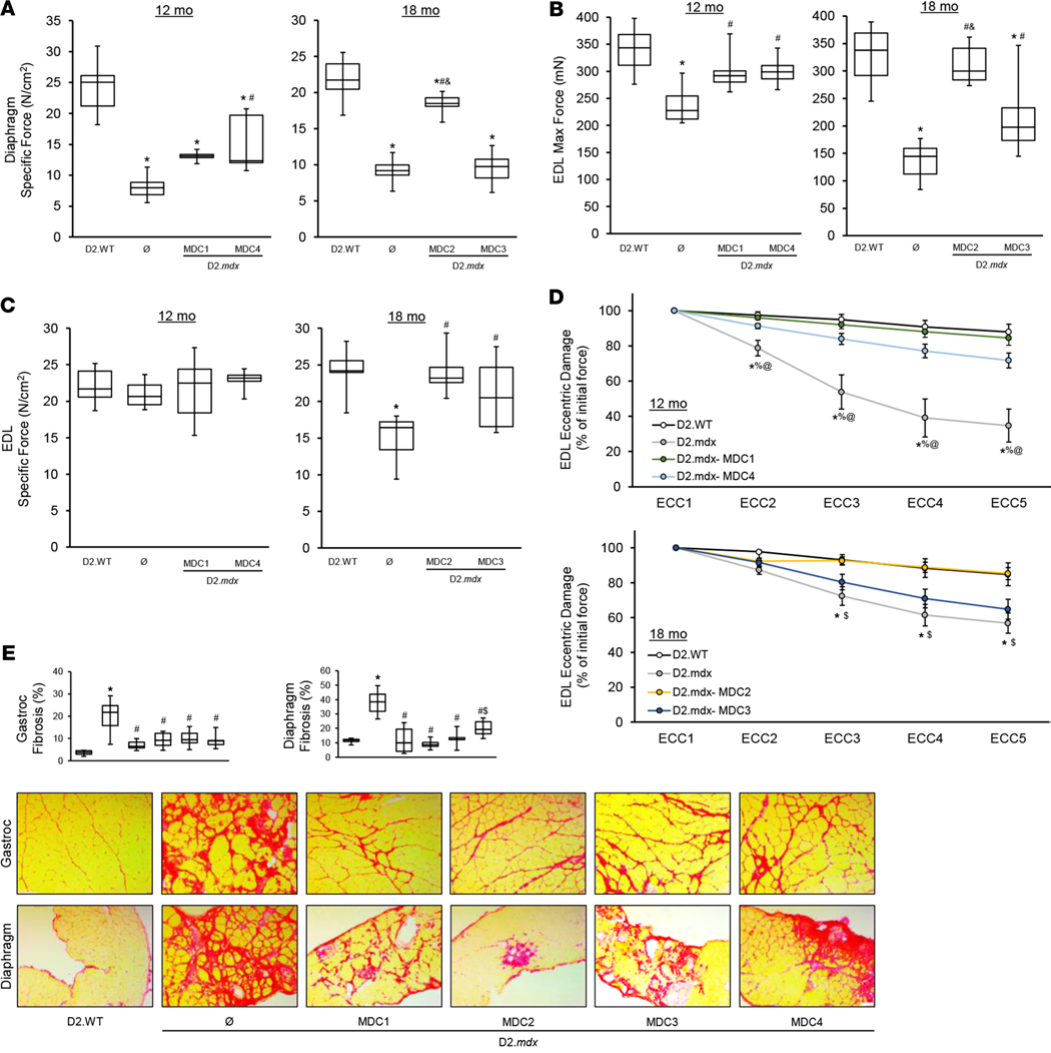
Microdystrophin provides partial rescue of D2.*mdx* skeletal muscle. Male D2.*mdx* mice were treated with microdystrophin (μDys) gene therapy at 1 month of age (refer to Figure 2A). (**A**–**D**) At the terminal endpoints of 12 and 18 months, ex vivo muscle function was performed for the diaphragm (**A**) and extensor digitorum longus muscles (EDL) (**B**–**D**) of D2.WT, untreated D2.*mdx*, and μDys-treated D2.*mdx* mice (*n* = 6–10). (**E**) Representative PSR-stained images of the gastrocnemius and diaphragm muscles with accompanying fibrosis quantifications for these groups. Scale bar: 75μm. Data were analyzed using 1-way ANOVA with Tukey HSD post hoc tests (α = 0.05) and displayed as box-and-whisker plots (**A**–**C** and **E**) (boxes indicate second and third quartiles, and error bars represent the minimum and maximum values) or mean ± SEM. *P < 0.05 compared with WT; ^#^*P* < 0.05 compared with untreated D2.*mdx*; ^%^*P* < 0.05 compared with MDC1; ^$^*P* < 0.05 compared with MDC2; ^&^*P* < 0.05 compared with MDC3; ^@^*P* < 0.05 compared with MDC4.

At terminal endpoint, ex vivo functional evaluations of diaphragm and extensor digitorum longus (EDL) muscles were performed. As anticipated by previous reports (10, 24, 25), microdystrophin treatment improved several features of skeletal muscle function, including increases in diaphragm-specific tension, EDL-specific tension, and EDL resistance to eccentric contraction-induced functional deficits, compared with untreated D2.*mdx* mice (Figure 3, A–D). However, these functional improvements were, for the most part, significantly diminished compared with D2.WT values. One of the clinical constructs, MDC3, provided much less benefit to the skeletal muscle than the other 3 constructs (Figure 3, A–D). In agreement with a partial skeletal muscle rescue by microdystrophin, the diaphragms of treated mice exhibited fibrotic lesions, albeit less than untreated D2.*mdx* animals (Figure 3E). Additionally, all MDCs significantly reduced fibrosis in the gastrocnemius (Figure 3E). Systemic microdystrophin gene therapy provides significant, albeit incomplete, rescue of D2.*mdx* skeletal muscle. The resulting phenotype appears to lie within the spectrum of a BMD-like disease, which likely represents an approximate ceiling of what would be expected of microdystrophin’s efficacy in the clinic.

### Microdystrophin gene therapy may not benefit D2.mdx hearts.

**Figure 4:**
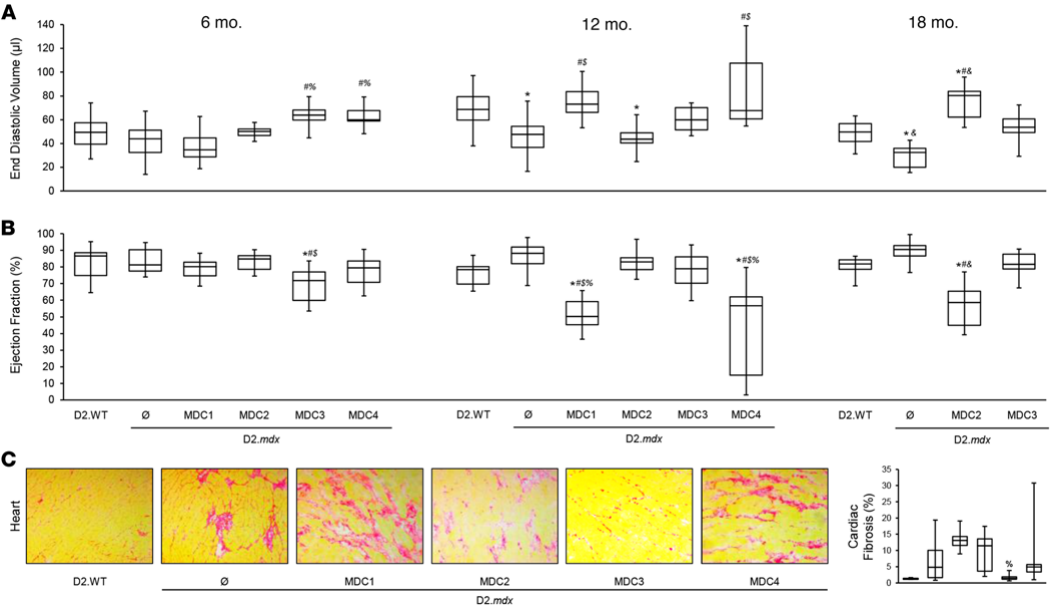
Long-term microdystrophin expression causes cardiomyopathy in D2.*mdx* mice. Male D2.*mdx* mice were treated with microdystrophin (μDys) gene therapy at 1 month of age (refer to Figure 2A). (**A** and **B**) End diastolic volume (**A**) and ejection fraction (**B**) were measured in D2.WT, untreated D2.*mdx*, and μDys-treated D2.*mdx* mice at 6, 12, and 18 months of age. (**C**) Representative PSR-stained images of the heart with accompanying fibrosis quantifications for these groups. Scale bar: 75μm. Data were analyzed using 1-way ANOVA with Tukey HSD post hoc tests (α = 0.05) and displayed as box-and-whisker plots (**A**–**C**) (boxes indicate second and third quartiles, and error bars represent the minimum and maximum values). *P < 0.05 versus D2.WT values; ^#^*P* < 0.05 versus D2.*mdx* values; ^%^*P* < 0.05 versus MDC1 values; ^$^*P* < 0.05 versus MDC2 values; ^&^*P* < 0.05 versus MDC3 values.

During the course of this study, longitudinal changes in cardiac function were assessed by collecting electrocardiograms and echocardiograms of all treatment groups at 6 and 12 months of age as well as additional 18-month measurements for mice that received MDCs 2 or 3. At 6 months of age, untreated D2.*mdx* hearts do not exhibit significant differences in function from D2.WT hearts; however, mice treated with MDCs 3 or 4 have increased left ventricular chambers (end diastolic volume) (Figure 4A), and this was accompanied by a decrease in ejection fraction in mice treated with MDC3 (Figure 4B). Mice that received MDC1 have a reduced stroke volume (SV) and subsequent reduction in cardiac output (CO) at 6 months of age (Supplemental Table 1).

By 12 months of age, D2.*mdx* mice exhibit cardiac dysfunction: left ventricular restriction as evidenced by a decrease in end diastolic volume (EDV; Figure 4A) that results in a decrease in SV and CO (Supplemental Table 2). Other parameters of diastolic dysfunction exhibited by D2.*mdx* mice include an elevated isovolumic relaxation time (IVRT), a decreased mitral valve early (MV E) velocity, and an impaired myocardial performance index (MPI; Supplemental Table 2). Likewise, mice treated with MDC2 display left ventricular restriction (a decrease in EDV; Figure 4A) that results in a decrease in SV and CO (Supplemental Table 2). In contrast, animals treated with either MDC1 or MDC4 dilate at 12 months of age (increase in EDV; Figure 4A) and have a significant decrease in ejection fraction (EF; Figure 4B). Additionally, a subset of MDC1-treated animals exhibits no discernable MV atrial (A) wave, visualized with pulsed-wave Doppler, have a compensatory increase in MV E and, therefore, have an elevated E/A ratio (Supplemental Table 2). In agreement with their profound effect on survival (Figure 2B), MDC1 and MDC4 induce severe cardiomyopathy with features of DCM.

By 18 months of age, MDC2-treated animals developed a DCM characterized by dilation (Figure 4A) and decreased systolic function (Figure 4B). In contrast, 18-month-old MDC3-treated animals have a sustained EF (Figure 4B) and a normalized EDV (Figure 4A). Unlike the findings in the gastrocnemius and diaphragm (Figure 3E), cardiac fibrosis was differentially affected by MDC treatments. MDC3-treated animals did not develop a DCM and had reduced cardiac fibrosis (Figure 4C). While MDC3 had the least effect on skeletal muscle, it best protected the heart in this study (Figure 4 and Supplemental Table 3). Moreover, none of the MDCs were able to correct all of the electrocardiogram abnormalities observed in D2.*mdx* mice (Supplemental Figure 2 and Supplemental Tables 4–6).

When assessing 2 of the MDCs with differential effects on the heart at 12 months of age, MDC2 and MDC4, we found that each construct had a different effect on cardiomyocyte calcium transients. Consistent with known Ca^2+^ overload signaling in DMD, D2.*mdx* cardiomyocytes exhibited elevated Ca^2+^ levels, and MDC2 normalized peak Ca^2+^ release and percentage of sarcomere length shortening (Supplemental Figure 5). In contrast, MDC4 exacerbates peak Ca^2+^ release without any normalization in contractility, potentially contributing to heart failure and premature death observed in MDC4-treated D2.*mdx* mice. Collectively, these data indicate that AAV-microdystrophin treatment could have a detrimental effect on the heart, depending on the microdystrophin design and expression levels.

### Potential mechanisms contributing to microdystrophin-induced cardiomyopathy.

We sought to explore potential mechanisms contributing to these detrimental cardiac outcomes. These investigations have led us to suspect 2 potential causes of this microdystrophin-induced cardiomyopathy: (a) microdystrophin competes with and displaces endogenously expressed utrophin at the cardiomyocyte sarcolemma and (b) the long-term overexpression of microdystrophin protein contributes to overload of the ubiquitin-proteosomal system (UPS), resulting in impairments in cardiomyocyte protein quality control. We present the data and observations in support of the first mechanism (utrophin displacement) as the main contributor to microdystrophin-induced acceleration of cardiomyopathy and to overload of the UPS occurring if the expression levels are high enough (as with MDC1).

**Figure 5:**
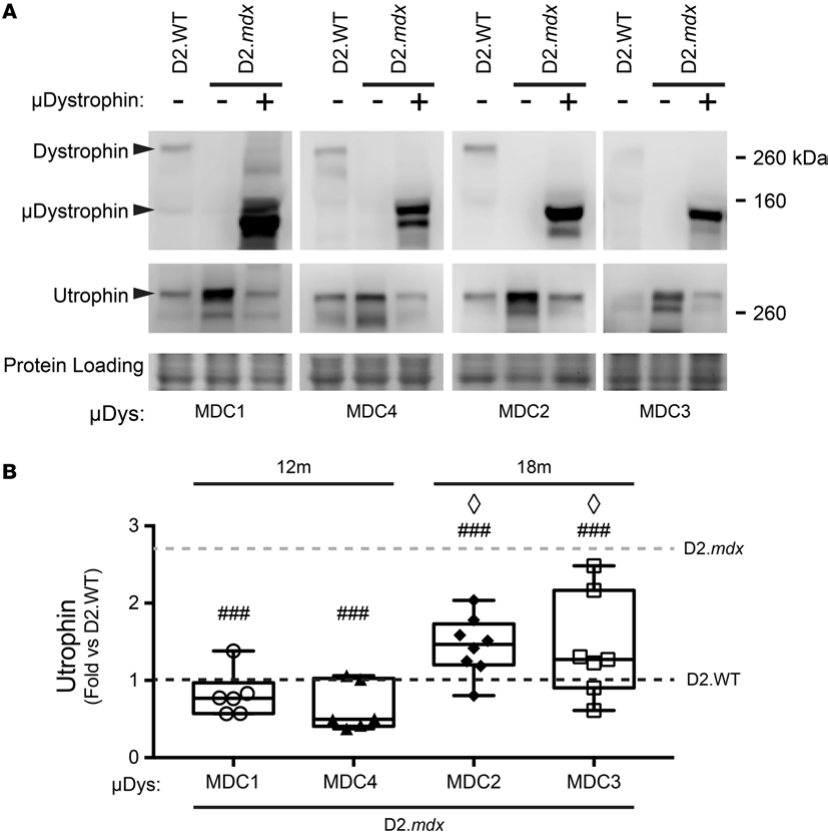
Diminution of sarcolemmal utrophin in microdystrophin overexpressing hearts. (**A**) Western blots of plasma membrane-enriched heart samples reveal an approximately 2- to 3-fold upregulation of membrane-associated utrophin in D2.*mdx* (*n* = 16, gray dotted line). (**B**) This increased membrane-associated utrophin was normalized to D2.WT levels (black dotted line) in the heart upon AAV-mediated overexpression of MDC2 (*n* = 8) or MDC4 (*n* = 7) and even reduced to approximately 60% of the D2.WTs upon over-expression of MDC1 (*n* = 6) or MDC4 (*n* = 6) (1-way ANOVA; ^###^*P* < 0.001 versus D2.*mdx*, ^◊^*P* < 0.05 versus D2.*mdx* + MDC4, Tukey post hoc comparison). Box-and-Whisker plots represent minimum-to-maximum, with second and third quartiles within the box and a line that indicates the mean.

The heart normally expresses a combination of utrophin and dystrophin, with potential overlapping and distinct roles that have yet to be elucidated. The ability of these 2 orthologous proteins to link the cytoskeleton to the extracellular matrix through their interactions with common partners is consistent with some degree of functional redundancy. Indeed, utrophin protein levels in the heart increase in the absence of dystrophin (26–28), and the removal of utrophin worsens the cardiac phenotype in the B10.*mdx* mice (29–31), with the total removal of utrophin being worse than haploinsufficiency. Thus, it is clear that utrophin can partially mitigate the loss of dystrophin. To potentially explain how high levels of microdystrophin leads to cardiomyopathy, we sought to determine if microdystrophin displaces utrophin from the cardiomyocyte membrane, as it is possible that strong overexpression of microdystrophin may phenocopy utrophin ablation via replacement with a truncated, and potentially less functional, dystrophin molecule. Therefore, we assessed the relative amounts of utrophin at the cardiac membrane by immunoblotting of membrane-enriched fractions of cardiac extracts from D2.WT, untreated D2.*mdx,* and microdystrophin-treated D2.*mdx* mice, in order to discern whether microdystrophin reduces membrane-associated utrophin in D2.*mdx*. The hearts of D2.*mdx* mice treated with either the MDC1 or the MDC4 microdystrophin exhibited significant decreases in utrophin immunoreactivity at the membrane to ~60% of D2.WT levels and ~30% of D2.*mdx* levels (Figure 5, A and B). In contrast, neither the MDC2 nor MDC3 microdystrophin displaced utrophin to the same extent.

This potential for microdystrophins to outcompete utrophin for association with the sarcolemma is not restricted to cardiomyocytes: AAV-mediated MDC4 expression in D2.*mdx* skeletal muscle also resulted in utrophin displacement from muscle fiber sarcolemma (Supplemental Figure 4A). Microdystrophin and utrophin thus appear to display a complementary and mutually exclusive pattern of expression in both heart and skeletal muscles of microdystrophin-treated D2.*mdx* mice. This likely results from competition between the 2 proteins for common binding partners present within the sarcolemma. There are 2 sites in WT skeletal muscle fibers where utrophin, along with dystrophin, accumulates at high density: the neuromuscular junction (NMJ) and the myotendinous junction (MTJ). Utrophin accumulation at these specialized portions of myofibers appear unperturbed despite overexpression of microdystrophin (Supplemental Figure 4, B and C). The absence of any noticeable utrophin depletion by microdystrophins at NMJs could result from the assembly of specialized subregions of the postsynaptic apparatus in which dystrophin (along with voltage-gated sodium channels) and utrophin (together with nicotinic acetylcholine receptors [nAChRs]) are spatially segregated (32–37). Such organization suggests distinct interactions that recruit dystrophin and utrophin to their respective domains with specificity. The degree of microdystrophin overexpression in skeletal muscle achieved in these experiments (approximately 10-fold lower than in the heart) may be insufficient to overcome utrophin’s affinity to its interacting proteins at the NMJs. Alternatively, the sheer density of utrophin at NMJs and MTJs that appears to far exceed the extrajunctional sarcolemma (Supplemental Figure 4, B and C) makes competition from microdystrophin less effective at these specialized membrane structures. In the case of NMJs, the density of nAChRs at the NMJs is measured to be up to 1,000-fold greater than at the extrasynaptic portions of the myofiber surface (38). While it is unknown whether utrophin levels at NMJs reach those of the nAChRs, its concentration at the synapse and its potential to form protein interactions distinct from those of dystrophin likely help maintain high-density synaptic accumulation of utrophin despite microdystrophin overexpression.

Another potential mechanism by which microdystrophin expression leads to cardiomyopathy, potentially in combination with utrophin displacement, is the saturation of the UPS by the excess microdystrophin molecules. Postmitotic cells, including cardiomyocytes, are especially susceptible to proteotoxicity stemming from accumulation of misfolded proteins, and impaired cardiomyocyte protein homeostasis has been shown to cause DCM-like cardiac phenotypes (39, 40). The sheer degree of overexpression (~50-fold higher than endogenous dystrophin of WT hearts) may saturate the capacity of the cardiomyocytes to ensure that proteins maintain their functional conformation and to breakdown/recycle those that are misfolded or damaged. Accumulation of polyubiquitinated proteins can serve as a molecular signature for UPS saturation and can lead to cardiomyopathy by impairing both the proper clearing of damaged/misfolded proteins and the timely turnover of typically short-lived proteins with specific signaling or transcriptional roles (41, 42). The AAV-mediated treatment of D2.*mdx* mice with the 4-repeat microdystrophin (MDC1) whose overexpression far exceeds the levels achieved by the 5-repeat variants (Figure 2C) produced a significant increase (~3-fold versus untreated D2.*mdx*) in the accumulation of K48 linkage-specific polyubiquitinated protein in the hearts (Supplemental Figure 3). Although, these findings do not conclusively establish disrupted protein homeostasis in cardiomyocytes as a major cause of DCM in D2.*mdx* hearts, the data presented are consistent with the hypothesis that impaired protein quality check in cardiomyocytes may accelerate the progression toward heart failure coupled with another contributing disease mechanism.

## Discussion

Microdystrophin gene therapy clinical trials are currently underway for the treatment of DMD. In the current report, we sought to critically examine the long-term efficacy of 4 different microdystrophin gene therapies using a severe mouse model of DMD to better understand the effect and potential limitations of these emerging therapeutics for the treatment of DMD. Previously, we demonstrated that the DBA/2J background strain does not exhibit an inherent cardiomyopathy (43), validating it as a useful background strain for this study. While there are numerous preclinical publications evaluating the efficacy of systemic AAV-mediated delivery of microdystrophin, many of these studies did not assess cardiac function (25, 44–46). Of the studies that did evaluate cardiac function (via EKG and pressure-volume catheters), a lower AAV dose than was used in the current study (and current clinical trials) was used, and/or the short study length would have prevented observing a progression to heart failure (24, 47–50). Therefore, to our knowledge, this is the first study that has assessed the long-term cardiac function of a severe mouse model of DMD following microdystrophin gene therapy using the high dose of AAV being used in clinical trials, albeit with a promoter that likely is stronger in the heart than those used in 2 of the clinical trials (51). A summary of our findings can be found in Table 1.

The dose of AAV (2 × 10^14^ gc/kg) used for this study was chosen to mirror doses being used in ongoing clinical trials with AAV-microdystrophin in patients with DMD (23). Clinical implementation of this dose has been dictated by the attempt to transduce as many skeletal muscle fibers as possible, which is assessed by postinjection muscle biopsies (23). There has been no consideration, however, of what this dose escalation may mean for the heart, and adequate modeling of these high doses and their long-term effect on the heart has not been previously performed. Furthermore, this work demonstrates that promoters that drive high-level expression of the transgene in skeletal muscles are desirable, but lower-level expression in the heart is needed. Fortunately, it appears that the only trial using CK8, which is strong in skeletal muscle and in the heart, is using a microdystrophin, MDC2, that is tolerated at higher expression levels in the heart. The degree of cardiac expression achieved in preclinical models and patients with DMD is dependent on the efficiency of cardiac muscle infection of the AAV capsid serotype used and the strength of the promoter in the heart. Based on the differential amounts of transgene expression we (Figure 2C) and others (46) have noted between murine skeletal and cardiac muscles, it is reasonable to assume that the heart is receiving more vector per cell than the skeletal muscle fibers (52). All of the promoters being used in the clinical trials were optimized for expression in both muscle types in mice, but the levels of expression in the human heart are unclear. The MHCK7 promotor driving MDC1 in clinical trials has been shown to have much greater expression in hearts than in skeletal muscle in mice (46, 53). Indeed, this promotor was said to be chosen for its high cardiac expression (23); however, the α myosin heavy chain enhancer (*Myh6*) that drives the high expression in mouse hearts will not achieve this in humans, since α myosin heavy chain is not expressed in human ventricles (54). α Myosin heavy chain is highly expressed in human atria (54); however, and it is currently unknown how this expression pattern will affect conductivity or atrial function in humans. An update on one of the clinical trials reported promising gene therapy transduction and microdystrophin expression in the skeletal muscles of trial participants 1 year following treatment (23). This microdystrophin has since received conditional FDA approval. However, the level of cardiac microdystrophin expression that is being achieved in patients with DMD remains unknown.

### Microdystrophin partially rescues skeletal muscle disease in D2.mdx mice.

The potential to modify a severe DMD disease using a truncated dystrophin molecule was initially suggested by the existence of mildly progressing patients with BMD who express mutant dystrophin proteins missing most of the rod domain (55, 56). Therefore, the ultimate goal of microdystrophin gene therapy is to convert DMD into a milder disease. Herein, we identified that long-term treatment of D2.*mdx* mice with AAV-packaged microdystrophins that are similar to the 3 clinical versions results in widespread transduction of the skeletal muscle and slowing of, but not halting, the progression of skeletal muscle disease. The treated muscles exhibit a slower progressing muscular degenerative disease, suggesting a conversion from DMD to a BMD-like pathology. Indeed, we find that the long-term trajectory of the skeletal muscle phenotype of microdystrophin-treated D2.*mdx* mice does represent a milder dystrophy, with progressive pathology most notable in the diaphragm. This progressive myopathy does not appear to be due to loss of microdystrophin in the mice over time (Figure 2D, bottom panel), as we initially anticipated, but rather is caused by the failure of microdystrophin to rescue all functions of full-length dystrophin, as in BMD.

It is likely that different designs of microdystrophin may slow the skeletal muscle disease to varying degrees; we see a less robust rescue of the skeletal muscles with MDC3 as compared with MDC1, MDC2, and MDC4. The microdystrophin MDC2 construct is able to restore neuronal nitric oxide synthase (nNOS) to the skeletal muscle membrane (24, 25), and nNOS localization may provide additional benefits to the skeletal muscle beyond sarcolemmal stability. Indeed, the diaphragm appears to be better rescued by this microdystrophin than by MDC3 (Figure 3A). This same region does not bind nNOS in the heart (57) but may serve other functions in the heart (58) and may have provided benefit that delayed the onset of DCM in the treated hearts, even though there was no effect on the onset of diastolic dysfunction (Figure 4A). On the other hand, MDC1 may exhibit increased membrane binding in the heart by the inclusion of repeats 1, 2, and 3, and this binding may enhance membrane localization and functional stability of the microdystrophin protein (59). Attempts to restore some or all of the missing C-terminus in order to better reconstitute the membrane complex may also improve function and further slow disease progression. However, it is becoming increasingly clear that all regions of dystrophin serve specific roles; thus, any microdystrophin is likely to be a physiological compromise as compared with full-length dystrophin and, potentially, utrophin. Only animal models that recapitulate aspects of the human disease, such as the D2.*mdx* mouse, can reveal which compromises are likely the most efficacious for dystrophic muscle. Ultimately ,it is likely that other types of therapies will need to be combined with microdystrophin gene therapy for the optimal management of DMD.

### Microdystrophin overexpression can cause cardiomyopathy.

Surprisingly, the clinical dose of 2 of the AAV-microdystrophins tested resulted in the development of a severe and early-onset life-limiting dilated cardiomyopathic failure. The very different cardiac outcome despite similar effects in the skeletal muscle does not appear to be due to the function of the microdystrophin. The premature onset of this cardiomyopathy appears to be related to the extent of microdystrophin overexpression in the heart and the specific design of the microdystrophin that alters its competition with utrophin for binding to the DGC. For instance, despite similar expression levels between MCD2 and MCD4, the latter both hastens onset of cardiomyopathy and displaces utrophin to a larger extent. We provide evidence that microdystrophin expression at the levels achieved with the CK8 promoter via high-dose AAV delivery causes displacement of native utrophin protein at the cardiomyocyte sarcolemma (dependent on microdystrophin design). The acceleration of the cardiomyopathy is coincident with the efficient displacement of utrophin by 2 of the microdystrophins (MDC1 and MDC4). How well a specific microdystrophin functionally substitutes for utrophin or full-length dystrophin in the heart will depend on which regions are in the microdystrophin and which regions are most critical for proper cardiac function. Competition will likely depend not only on the degree of overexpression but also on the design of the microdystrophin and its effect on binding partners, such as sytrophins, dystropbrevin, cavins, cryab, cypher, and ahnak1 (57).

A recent study (60) demonstrated that microdystrophin is beneficial to the heart in the total absence of utrophin in a B10.*mdx* background. However, comparing that study with this study is difficult since the utrophin was missing from the heart throughout development, possibly allowing adaptations that cannot occur with acute postnatal displacement of utrophin by overexpression of microdystrophin. In the absence of utrophin, we would predict that all microdystrophin constructs examined in this study should slow the onset of cardiac dysfunction and failure as compared with no intervention.

In contrast to our demonstration of microdystrophin outcompeting utrophin along the skeletal muscle fibers of the D2.*mdx* mice, but not at the neuromuscular and MTJs (Supplemental Figure 4A), the study from Krishna et al. (61) was interpreted as demonstrating that microdystrophin does not compete with utrophin. This was based on their observation that AAV-delivered microdystrophin colocalizes with utrophin along the fibers in skeletal muscle. However, their observation was in a mouse that had higher levels of utrophin and lower levels of microdystrophin along the muscle fibers compared with our case. They used dystrophin-deficient transgenic mice that expressed higher than normal levels of utrophin from all skeletal muscle nuclei and received about 10-fold less of an AAV-CK8-microdystrophin dose compared with our mice, shifting the competitive advantage to utrophin.

The higher overexpression of a 4-repeat microdystrophin (MDC1) also produced evidence that suggests impaired protein quality check in cardiomyocytes. High level of transgene overexpression, in and of itself*,* can be detrimental if the increased protein turnover overloads the protein breakdown capacity of the cell (39) and likely puts an extra energetic load on an already stressed heart (62). Indeed, it was previously shown that 100-fold transgenic overexpression of a minidystrophin was associated with cardiac toxicity (63). This minidystrophin is likely more efficacious in the heart than any microdystrophin, and this may allow higher levels of overexpression to be tolerated.

Our current data highlight the benefits, limitations, and potential deleterious consequences of maximizing microdystrophin overexpression in both skeletal and cardiac muscle for the treatment of DMD. Likely, all microdystrophin constructs would show some benefit in the heart if transgene expression levels are kept closer to physiological dystrophin levels to avoid pathological side effects that may include utrophin displacement or overload of the UPS.

### Conclusion.

Whether or not the patients with DMD currently being dosed with AAV-microdystrophin in clinical trials are at risk of accelerated cardiac disease is unclear. It may be years before this question can be addressed, given that it requires 8–12 months to clearly see this cardiomyopathy development in mice. However, our preclinical data in mice suggest that there is reason to be concerned that, while the skeletal muscles improve in individuals with DMD receiving the current AAV-microdystrophin vectors, the dystrophic hearts may not be improved by these treatments. Even if the treatment is modestly beneficial for the heart, the increased load on the heart due to the improved skeletal muscle function may accelerate the onset of DCM and heart failure. Therefore, frequent monitoring of the cardiac status of these patients should be performed and prophylactic use of cardioprotective drugs — including ACE inhibitors/angiotensin receptor blockers, beta blockers, and/or mineralocorticoid receptor antagonists — should be considered. If the observations in this study are recapitulated in patients with DMD, then (a) microdystrophins may need to be optimized for cardiac rescue and (b) delivery of microdystrophin to the heart may need to be dissociated from skeletal muscle via the use of promoters designed to drive less expression in the heart than in skeletal muscle.

## Methods

### Sex as a biological variable.

This study only involved the use of male mice, since DMD is an X-linked disease that primarily affects men.

### Animals.

This study used male D2.WT (The Jackson Laboratory, stock no. 000671) and D2.*mdx* (The Jackson Laboratory, stock no. 013141) mice from colonies originally obtained from The Jackson Laboratory. Mice were housed 1–5 mice per cage; randomly assigned into groups; provided ad libitum access to food (NIH-31 Open formulation diet; Envigo, 7917), water, and enrichment; and maintained on a 12-hour light/dark system.

### Microdystrophin constructs and vector production.

Codon-optimized microdystrophin was synthesized by Genscript and cloned into a pAAV shuttle plasmid containing the striated muscle-specific CK8 promoter (20) and a minimized synthetic polyadenylation signal sequence (64). AAV viral vector packaging was performed using the triple-transfection method, as previously described (21, 65).

### Ex vivo muscle function.

Maximal tetanic tension assessments of the EDL and diaphragm muscles were evaluated as previously described (66) by the University of Florida Physiological Assessment Core. Subsequently, a series of 5 eccentric contractions (stimulated at 80 Hz for 700 ms) with a stretch of 10% optimal length was imposed on the muscle in the last 200 ms of each contraction. Each contraction was separated by a 5-minute rest period. Following experimental procedures, muscles were weighed, frozen embedded in OCT or snap-frozen, and stored at –80°C until further use.

### Echocardiography and electrocardiograms.

Electrocardiograms and transthoracic echocardiograms were performed using the Vevo 3100 preclinical imaging system (Fujifilm Visualsonics). Mice were anesthetized using 3% isoflurane and maintained at 1.5%–2% to keep heart and respiration rates consistent among treatment groups. Body temperature was maintained at 37°C throughout imaging. Electrocardiograms were imported into LabCharts (ADInstruments) for analysis. Four images were acquired for each animal: B-mode parasternal long axis (LAX), B-mode short axis (SAX), M-mode SAX, and apical 4-chamber view with color Doppler and pulsed-wave Doppler. M-mode SAX images were acquired at the level of the papillary muscle. Flow through the mitral valve was sampled at the point of highest velocity, as indicated by aliasing, with the pulsed-wave angle matching the direction of flow. Images were imported into Vevo LAB for analysis. Measurements of M-mode SAX and pulsed-wave Doppler images were made from 3 consecutive cardiac cycles between respirations.

### Fractionation, protein extraction, and immunoblotting.

Snap-frozen mouse heart and gastrocnemius muscles were finely crushed and homogenized in a phosphate-based homogenization solution — 2 mM sodium phosphate, 80 mM NaCl, 1 mM EDTA (67) — supplemented with 1 mM phosphatase/protease inhibitor cocktail (PMSF; Thermo Fisher Scientific), and centrifuged at 12,000*g* for 10 minutes at 4°C. The supernatant (soluble cytosolic fraction) was collected. The pelleted noncytosolic (including membrane and cytoskeletal) fraction was then resuspended in the extraction buffer — homogenization solution supplemented with the following: 20 μg/ml DNase I (MilliporeSigma), 10 μM Vinblastine (Caymen Chemicals), 100 mM Swinholide A (Caymen Chemicals), 100 mM Mycalolide B (Focus Biomolecules), 1% Digitonin (Biosynthe), 0.5% NP-40, 1% SDS — and extracted on ice for 45 minutes with occasional vortexing, followed by a 15-minute incubation at 37°C. The insoluble fraction was pelleted by centrifugation at 12,000*g* for 10 minutes at 4°C, and soluble membrane fraction was collected. The protein concentration of soluble cytoplasmic and membrane fractions was determined using the Bio-Rad Protein Assay (Bio-Rad Laboratories). Samples were boiled in 4× sample buffer, and proteins were separated using a 4%–12% SDS polyacrylamide gels (Thermo Fisher Scientific) and transferred to nitrocellulose membranes using the iBlot system (Invitrogen). Membranes were incubated at room temperature with 5% BSA-TBST and then overnight with primary antibodies at 4°C. Following the overnight primary antibody incubation, the membranes were washed with TBST, incubated with species-appropriate horseradish peroxidase–conjugated secondary antibody (Cell Signaling Technology), incubated with ECL reagent (Thermo Fisher Scientific), and imaged using the Li-Cor C-DiGit imaging system (Li-Cor Biosciences). Membranes were probed for GAPDH for cytosol/noncytosol fractionation and stained with Ponceau S to control for equal protein loading and for normalization. The following primary antibodies were used for immunoblotting in the present study: MANHINGE1B (1:100; clone 10F9; Developmental Studies Hybridoma Bank [DSHB]), MANEX1011B (1:100; clone 1C7; DSHB), MANEX1011C (1:100; clone 4F9; DSHB), utrophin-A (1:1000; ABN1739; EMD Millipore), Polyubiquitin (K48-linkage; 1:2000; 4389, Cell Signaling Technology), and GAPDH (1:2000; SC-25778; Santa Cruz Biotechnology Inc.). Band signal intensities were measured using Image Studio Lite software (Li-Cor Biosciences), normalized to sample loading (Ponceau S stain), and reported relative to respective control samples.

### Quantification of vector genomes.

DNA was isolated from crushed heart samples using the DNeasy Blood & Tissue Kit (Qiagen, 69506) following the manufacturer’s instructions. Real-time PCR was performed with 100 ng of DNA from each sample using QuantiTect SYBR Green PCR Kit (Qiagen, 204145). Primers used during this assay include those for codon optimized human microdystrophin (recognizes vector genomes; Forward: 5′- TGA CGC GTG GTA CCT CTA -3′; Reverse: 5′- GGA AGA TCC TAA TCG ATC ACA CA -3′) and a genomic DNA region in the *Rpl32* locus of murine chromosome 6 (recognizes diploid genomes; Forward: 5′- GAG AAG GTT CAA GGG CCA GAT -3′; Reverse: 5′- AGC TCC TTG ACA TTG TGG ACC- 3′). Vector genome content was quantitated normalized to diploid vector genome expression using the ΔΔCT method.

### Immunofluorescence and histological evaluations.

Fresh-frozen OCT-embedded hearts and gastrocnemius muscles were sectioned at 10 μm and fixed in ice-cold acetone. The sections were rehydrated in PBS, blocked in 5% BSA-PBS at room temperature, and incubated with primary antibodies overnight at 4°C. Mouse tissue sections to be incubated with mouse monoclonal antibodies were first incubated with a solution containing donkey anti–mouse IgG AffiniPure Fab fragments (1:25 in PBS; Jackson ImmunoResearch, 715-007-003) for 1 hour prior to blocking. Following PBS washes, sections were incubated at room temperature with species- and isotype-appropriate fluorescent dye–conjugated secondary antibodies and coverslipped using Prolong Gold anti-fade mounting medium (Thermo Fisher Scientific). The following primary antibodies were used for immunofluorescence in the present study: MANHINGE1B (1:100; clone 10F9; DSHB), MANEX1011B (1:100; clone 1C7; DSHB), MANDAG2 (1:100; clone 7A11; DSHB), utrophin-A (1:1000; ABN1739; EMD Millipore), utrophin (1:50; VP-U579; Vector Laboratories), Dystrobrevin (1:500; 610766; BD Biosciences); and Syntrophins (1:2000; 11425; Abcam). NMJs were identified using fluorescent dye–conjugated α-bungarotoxin (1:500; Thermo Fisher Scientific) to label nAchRs localized to the postsynaptic motor endplates. Image acquisition was performed with a Leica Application Suite X software on either a Leica TSC-8 confocal system or a Leica DMR epifluorescence microscope equipped with a Leica DCF480 digital camera. Comparative images were stained, imaged, and processed simultaneously under identical conditions.

Picrosirius red (PSR) staining was performed as previously described (13) following decalcification of muscle sections using Formical-2000 (StatLab). Slides were visualized with a Leica DMR microscope, and images were acquired using a Leica DFC310FX camera interfaced with Leica LAS X software. Images were processed and analyzed by investigators blinded to study groups using ImageJ software (NIH).

### Calcium kinetics.

Harvested hearts were placed in a Langendorff setup, perfused with a Type II Collagenase (Worthington) and Protease (MilliporeSigma) digestion buffer, and enzymatically digested. Cells were released by mechanical means, filtered via 200 μm mesh filters, and spun down for further separation. The pellet of cell pellets were plated and stepped up with Ca^2+^ to 1 mM over 30–45 minutes and were loaded with Fura-2AM Ca^2+^ *dye*. After reaching 1 mM Ca^2+^, cells were assessed for simultaneous Ca^2+^ transients and sarcomere length shortening using an IonOptix CnC System (IonOptix) and analyzed using CytoSolver software (IonOptix).

### Statistics.

Statistical analysis was performed using unpaired, 2-tailed Welch’s *t* test (α = 0.05), ANOVA (1-way, 2-way, or repeated measures) followed by Tukey HSD post hoc tests (α = 0.05), and Kaplan-Meier estimator analyses (α = 0.05), where appropriate. A *P* value less than 0.05 was considered significant. Data are displayed as mean ± SEM, box-and-whisker plots, or survival curves.

### Study approval.

All animal studies were approved and conducted in accordance with the University of Florida IACUC.

### Data availability.

The data sets generated during and/or analyzed during the current study are available in the Supporting Data Values file.

## Author contributions

Study design was contributed by CCH, YL, DWH, and HLS. Experimental procedures and data acquisition were conducted by CCH, YL, BLL, and DWH. Essential reagents were produced and provided by JX and GG. All authors were involved in data analysis, interpretation, data presentation, and manuscript writing.

## Supplementary Material

Supplemental data

Unedited blot and gel images

Supporting data values

## Figures and Tables

**Table 1:**
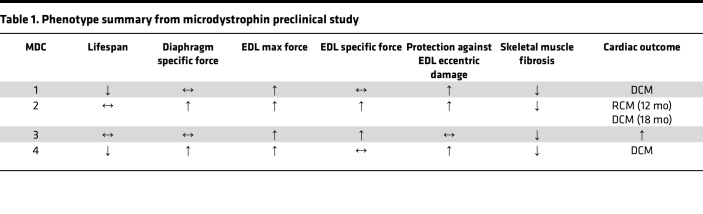
Phenotype summary from microdystrophin preclinical study

## Version 1 05/07/2024

In-Press Preview

## Version 2 06/10/2024

Electronic publication
