# Supplementary material for: Potential limitations of microdystrophin gene therapy for Duchenne muscular dystrophy
Source: JCI Insight. 2024 May 7;9(11):e165869. doi: 10.1172/jci.insight.165869 (PMC11382885; doi:10.1172/jci.insight.165869)
Supplement: Supplemental data [file jciinsight-9-165869-s128.pdf]

SUPPLEMENTAL MATERIALS

SUPPLEMENTAL TABLES

		D2.WT	D2.*mdx*	MDC1	MDC2	MDC3	MDC4
LV Structure and Function	**HR (BPM)**	442.73±12.55	405.16±11.12	418.11±15.39	423.23±12.55	396.57±14.09	422.44±14.46
	**EDD (mm)**	3.44±0.11	3.18±0.15$^{\&@}$	3.04±0.14$^{\&@}$	3.46±0.04	3.83±0.09	3.79±0.08
	**ESD (mm)**	1.68±0.15	1.54±0.14	1.61±010	1.68±0.06	2.34±0.15*$^{\#\%\$}$	2.04±0.13
	**EDV (µl)**	49.90±3.78	42.00±4.21$^{\&@}$	37.43±4.27$^{\&@}$	49.67±1.49	63.61±3.57	61.97±3.03
	**ESV (µl)**	9.77±2.10	7.66±1.47	7.80±1.20	8.40±0.80	20.10±3.07	14.29±2.13
	**EF (%)**	82.48±2.70	83.46±2.23	79.25±2.06	83.07±1.58	69.39±3.79*$^{\#\$}$	77.10±3.31
	**FS (%)**	52.02±3.04	52.46±2.75	47.00±2.08	51.45±1.78	39.26±3.06*$^{\#\$}$	46.25±3.27
	**SV (µl)**	40.13±2.07	34.34±3.00$^{@}$	29.62±3.39*$^{\$\&@}$	41.27±1.47	43.51±2.36	47.68±2.92
	**CO (ml/min)**	17.78±1.05	13.78±1.10	12.04±1.10*$^{\$@}$	17.52±0.90	16.57±1.60	19.95±0.94$^{\#}$
	**LV Mass (mg)**	183.39±13.995	145.95±13.46	142.53±9.73	146.44±7.03	137.74±10.67	215.65±14.30$^{\#\%\$\&}$
	**SAW (mm)**	2.10±0.10	1.94±0.13	1.98±0.09	2.06±0.10	1.66±0.08*	2.03±0.09
	**DAW (mm)**	1.37±0.07	1.30±0.12	1.46±0.10	1.34±0.05	1.15±0.06	1.30±0.07
	**SPW (mm)**	2.03±0.12	1.87±0.12	1.84±0.07	1.70±0.08	1.49±0.09*$^{@}$	2.13±0.07$^{\$}$
	**DPW (mm)**	1.54±0.11	1.39±0.15	1.31±0.08	1.18±0.06	1.03±0.07*$^{@}$	1.67±0.11$^{\$}$
Mitral Inflow	**NFT (ms)**	73.41±2.01	77.79±2.99	76.90±1.83	67.79±1.47	79.33±2.73	73.99±2.47
	**AET (ms)**	47.36±1.21	45.70±2.13	47.91±0.80	44.89±0.80	49.88±1.10	45.06±1.60
	**IVCT (ms)**	8.67±0.75	12.59±1.23*	11.80±0.87	9.59±0.71	10.71±0.71	10.63±1.10
	**IVRT (ms)**	17.78±1.28	20.73±1.45	17.51±1.12	14.27±0.82$^{\#\&}$	19.47±1.17	18.58±0.69
	**MV E(mm/s)**	700.82±23.34	582.98±49.51	789.13±35.81$^{\#@}$	772.18±10.48$^{\#@}$	715.89±24.87	556.78±44.33
	**MV A (mm/s)**	566.15±20.50	423.34±28.36	240.80±80.93*$^{\#\$\&@}$	554.40±9.08	506.99±18.76	434.33±29.96
	**MV E/A**	1.25±0.03	1.37±0.07	1.50±0.13	1.40±0.04	1.43±0.08	1.28±0.04
	**LV MPI IV**	0.60±0.05	0.75±0.05$^{\&}$	0.61±0.03	0.53±0.03	0.60±0.03	0.65±0.03

Supplemental Table 1: Cardiac function at 6 months of age. HR-heart rate; EDD-end diastolic diameter; ESD-end diastolic diameter; EDV-end diastolic volume; EF-ejection fraction; FS-fractional shortening; SV-stroke volume; CO-cardiac output; SAW-systolic anterior wall; DAW-diastolic anterior wall; SPW-systolic posterior wall; DPW-diastolic posterior wall; NFT-non-filling time; AET-aortic ejection time; IVCT-isovolumic contraction time; IVRT-isovolumic relaxation time; MV E-mitral valve early wave; MV A-mitral valve atrial wave; MPI-myocardial performance index (IVCT + IVRT)/AET; *p<0.05 compared to WT; $^{\#}$p<0.05 compared to untreated D2.*mdx*; $^{\%}$p<0.05 compared to MDC1; $^{\$}$p<0.05 compared to MDC2; $^{\&}$p<0.05 compared to MDC3; $^{@}$p<0.05 compared to MDC4.

		D2.WT	D2.*mdx*	MDC1	MDC2	MDC3	MDC4
LV Structure and Function	**HR (BPM)**	418.83±12.19	405.42±8.87	434.29±18.48	422.19±6.54	417.31±16.89	394.86±15.10
	**EDD (mm)**	3.95±0.11	3.16±0.13*	4.10±0.16$^{\#\$}$	3.31±0.13*	3.75±0.10	4.26±0.22$^{\#\$}$
	**ESD (mm)**	2.19±0.12	1.41±0.13*	3.04±0.21$^{\#\$\&}$	1.61±0.16	2.00±0.20	3.33±0.40*$^{\#\$\&}$
	**EDV (µl)**	69.06±4.2	41.49±3.96*	75.19±6.83$^{\#\$}$	45.25±4.30	60.57±3.84	83.66±10.37$^{\#\$}$
	**ESV (µl)**	17.23±2.18	6.49±1.35	37.58±5.98$^{\#}$	8.26±1.74	14.44±3.32	53.37±14.78*$^{\#\$\&}$
	**EF (%)**	76.26±1.89	86.31±2.21	51.44±4.44*$^{\#\$\&}$	83.18±2.57	77.81±4.06	44.69±9.36*$^{\#\$\&}$
	**FS (%)**	44.99±1.72	56.68±2.88*	26.28±2.75*$^{\#\$\&}$	52.17±3.41	47.28±4.04	23.80±5.35*$^{\#\$\&}$
	**SV (µl)**	51.83±2.43	35.00±2.96*	37.61±2.56	37.00±2.77*	46.13±1.34	30.29±5.22*$^{\&}$
	**CO (ml/min)**	21.47±0.88	14.15±1.19*	16.29±1.26	15.60±1.20*	19.16±0.63	12.10±2.26*$^{\&}$
	**LV Mass (mg)**	173.51±8.35	140.60±13.18	190.54±11.43	138.49±8.21	165.81±11.18	216.60±16.13$^{\#\$}$
	**SAW (mm)**	1.99±0.07	2.01±0.09	1.80±0.06	2.07±0.10	1.94±0.05	1.78±0.12
	**DAW (mm)**	1.29±0.05	1.28±0.05	1.32±0.05	1.39±0.09	1.39±0.03	1.40±0.50
	**SPW (mm)**	1.73±0.05	1.87±0.10	1.52±0.07	1.67±0.06	1.80±0.16	1.54±0.15
	**DPW (mm)**	1.20±0.04	1.33±0.10	1.22±0.08	1.15±0.06	1.15±0.09	1.28±0.10
Mitral Inflow	**NFT (ms)**	73.50±2.27	73.60±1.77	74.03±3.00	69.51±1.05	70.46±2.72	77.84±2.66
	**AET (ms)**	47.25±1.11	43.24±1.12	45.83±2.01	44.57±1.55	42.78±1.63	45.47±1.97
	**IVCT (ms)**	10.17±0.99	11.10±0.74	9.44±0.22	8.35±0.66	8.86±0.87	8.76±1.80
	**IVRT (ms)**	16.16±0.71	20.30±1.00*	15.97±1.32	16.48±1.18	19.02±0.91	24.43±1.52*$^{\%\$\&@}$
	**MV E (mm/s)**	723.97±16.54	590.88±25.41*$^{\%}$	773.06±90.47	649.64±27.95	705.68±30.56	559.36±20.02*$^{\%\&}$
	**MV A (mm/s)**	518.86±18.18	501.89±21.49	206.46±45.72*$^{\#\$\&@}$	523.31±24.63	512.44±28.29	502.69±25.70
	**MV E/A**	1.42±0.05	1.19±0.05	4.83±1.17*$^{\#\$\&@}$	1.26±0.08	1.39±0.06	1.14±0.05
	**LV MPI IV**	0.56±00.03	0.73±0.04*$^{\$}$	0.56±0.01	0.57±0.04	0.65±0.03	0.69±0.08

Supplemental Table 2: Cardiac function at 12 months of age. HR-heart rate; EDD-end diastolic diameter; ESD-end diastolic diameter; EDV-end diastolic volume; EF-ejection fraction; FS-fractional shortening; SV-stroke volume; CO-cardiac output; SAW-systolic anterior wall; DAW-diastolic anterior wall; SPW-systolic posterior wall; DPW-diastolic posterior wall; NFT-non-filling time; AET-aortic ejection time; IVCT-isovolumic contraction time; IVRT-isovolumic relaxation time; MV E-mitral valve early wave; MV A-mitral valve atrial wave; MPI-myocardial performance index (IVCT + IVRT)/AET; *$p<0.05$ compared to WT; $^{\#}p<0.05$ compared to untreated D2.*mdx*; $^{\%}p<0.05$ compared to MDC1; $^{\$}p<0.05$ compared to MDC2; $^{\&}p<0.05$ compared to MDC3; $^{@}p<0.05$ compared to MDC4.

		D2.WT	D2.*mdx*	MDC2	MDC3
**LV Structure and Function**	**HR (BPM)**	454.98±10.32	398.53±13.74*	416.89±10.37	423.58±13.48
	**EDD (mm)**	3.41±0.13	2.76±0.14*$^{\&}$	4.09±0.14*$^{\#\&}$	3.54±0.14
	**ESD (mm)**	1.76±0.11	1.09±0.16*$^{\&}$	2.89±0.23*$^{\#\&}$	1.77±0.16
	**EDV (µl)**	48.58±4.22	29.38±3.51*$^{\&}$	74.54±5.86*$^{\#\&}$	53.11±4.67
	**ESV (µl)**	9.78±1.69	3.44±0.95	34.05±6.23*$^{\#\&}$	10.27±2.28
	**EF (%)**	80.52±2.04	89.67±2.55	56.53±5.33*$^{\#\&}$	82.00±2.67
	**FS (%)**	48.51±1.94	61.63±4.66*	29.96±3.61*$^{\#\&}$	50.62±2.76
	**SV (µl)**	38.80±3.02	25.94±2.86*$^{\$\&}$	40.49±1.89	42.83±2.90
	**CO (ml/min)**	17.77±1.60	10.36±1.24*$^{\$\&}$	16.81±0.65	18.12±1.38
	**LV Mass (mg)**	150.68±10.19	119.52±9.72$^{\$\&}$	175.81±13.76	159.02±6.82
	**SAW (mm)**	2.01±0.06	2.09±0.09	1.76±0.10$^{\#}$	1.99±0.05
	**DAW (mm)**	1.31±0.06	1.36±0.05	1.31±0.08	1.33±0.05
	**SPW (mm)**	1.75±0.07	1.89±0.14	1.45±0.10$^{\#}$	1.84±0.10
	**DPW (mm)**	1.29±0.07	1.33±0.11	1.12±0.11	1.29±0.08
**Mitral Inflow**	**NFT (ms)**	70.38±2.33	79.93±3.31	76.24±2.72	69.97±2.09
	**AET (ms)**	42.67±1.40	42.57±1.71	46.07±1.98	42.57±1.67
	**IVCT (ms)**	9.82±1.72	13.11±1.41	10.92±0.76	8.69±0.99
	**IVRT (ms)**	18.45±1.30	24.82±1.55*$^{\$\&}$	19.49±0.81	19.28±0.91
	**MV E (mm/s)**	665.22±24.52	523.23±15.74*$^{\&}$	529.39±32.29	687.43±28.77*$^{\&}$
	**MV A (mm/s)**	503.36±30.73	504.48±28.00	428.33±29.94$^{\&}$	538.38±19.70
	**MV E/A**	1.34±0.07	1.06±0.06*	1.25±0.07	1.28±0.04
	**LV MPI IV**	0.67±0.04	0.90±0.06*$^{\$\&}$	0.67±0.04	0.67±0.05

Supplemental Table 3: Cardiac function at 18 months of age. HR-heart rate; EDD-end diastolic diameter; ESD-end diastolic diameter; EDV-end diastolic volume; EF-ejection fraction; FS-fractional shortening; SV-stroke volume; CO-cardiac output; SAW-systolic anterior wall; DAW-diastolic anterior wall; SPW-systolic posterior wall; DPW-diastolic posterior wall; NFT-non-filling time; AET-aortic ejection time; IVCT-isovolumic contraction time; IVRT-isovolumic relaxation time; MV E-mitral valve early wave; MV A-mitral valve atrial wave; MPI-myocardial performance index (IVCT + IVRT)/AET; *p<0.05 compared to WT; $^{\#}$p<0.05 compared to untreated D2.*mdx*; $^{\$}$p<0.05 compared to MDC2; $^{\&}$p<0.05 compared to MDC3.

	D2.WT	D2.*mdx*	MDC1	MDC2	MDC3	MDC4
**RR Interval (ms)**	139.19±4.01	151.59±5.58	146.12±5.93	143.87±5.24	150.86±5.14	143.13±6.07
**Heart Rate (BPM)**	435.36±11.58	400.44±14.06	416.46±16.02	421.55±13.81	400.64±12.35	425.32±16.23
**PR Interval (ms)**	36.12±0.95	35.84±0.85	35.59±1.66	34.50±0.69	33.72±0.66	33.71±1.13
**P Duration (ms)**	7.51±0.37	8.01±0.71	9.03±0.56	7.48±0.47	7.39±0.38	8.49±1.16
**QRS Interval (ms)**	10.37±0.37	10.30±0.50	11.49±0.23	9.75±0.52	10.73±0.69	10.05±0.19
**QT Interval (ms)**	16.63±0.47	19.51±0.46	22.68±1.85$^{*\$\&@}$	17.75±0.54	16.24±0.69	16.81±0.81
**QTc (ms)**	44.77±1.44	50.18±0.74	59.86±5.44$^{*\$\&@}$	46.98±1.66	41.81±1.48	44.67±2.32
**JT Interval (ms)**	6.27±0.60	9.21±0.51	11.63±1.97$^{*\&@}$	8.00±0.33	5.51±0.42	6.76±0.78
**T_{peak} T_{end} Interval (ms)**	3.96±0.40	6.31±0.76	7.33±1.75$^{*\&}$	5.27±0.35	3.86±0.40	4.64±0.64
**P Amplitude (V)**	117.02±10.37	104.16±8.59	150.75±12.94	138.51±8.97	122.42±16.02	118.91±11.78
**Q Amplitude (V)**	13.23±3.50	16.31±2.47	-69.43±36.84	10.68±2.54	-25.73±38.18	3.71±4.53
**R Amplitude (V)**	510.52±39.85	566.08±50.45	324.59±43.83$^{\#\&}$	502.82±43.06	575.96±58.94	454.29±29.51
**S Amplitude (V)**	-245.54±53.24	-412.47±121.80	-301.15±80.81	-212.01±67.75	-134.83±75.48	-365.07±65.86
**T Amplitude (V)**	214.28±26.84	318.25±35.20	170.69±30.02$^{\#}$	291.91±35.43	210.08±37.58	242.43±34.44

Supplemental Table 4: Electrocardiogram measures at 6 months of age. *p<0.05 compared to WT; $^{\#}$p<0.05 compared to untreated D2.*mdx*; $^{\%}$p<0.05 compared to MDC1; $^{\$}$p<0.05 compared to MDC2; $^{\&}$p<0.05 compared to MDC3; $^{@}$p<0.05 compared to MDC4.

	D2.WT	D2.*mdx*	MDC1	MDC2	MDC3	MDC4
**RR Interval (ms)**	139.30±3.99	140.84±3.31	138.53±6.59	148.43±3.52	141.50±5.40	153.38±6.82
**Heart Rate (BPM)**	432.77±11.83	427.63±10.08	437.28±17.69	405.76±9.34	427.73±16.37	396.16±16.17
**PR Interval (ms)**	33.10±1.13	34.84±0.62	37.30±1.02	35.68±0.85	32.81±1.42	39.24±3.21
**P Duration (ms)**	8.08±0.36	7.59±0.63	9.18±0.58	7.14±0.64	7.94±0.40	11.35±1.04$^{*\#\$\&}$
**QRS Interval (ms)**	10.21±0.79	10.24±0.44	11.18±0.22	10.76±0.45	11.66±0.97	12.39±0.88
**QT Interval (ms)**	16.93±0.46	20.82±0.68*	21.76±0.84*	21.45±0.75*	19.47±0.65	21.45±1.42*
**QTc (ms)**	45.40±1.00	55.61±2.12*	58.85±3.15*	55.71±1.84*	52.04±2.44	54.81±3.18
**JT Interval (ms)**	6.72±0.69	10.59±0.65*	10.58±0.73*	10.69±0.56*	7.81±0.49	9.06±0.95
**T_{peak} T_{end} Interval (ms)**	4.70±0.65	7.06±0.63	8.28±0.76*	7.49±0.88	5.61±0.34	6.54±1.06
**P Amplitude (V)**	112.74±4.97	103.85±13.64	134.12±10.08	82.09±11.18	108.49±16.63	106.79±14.17
**Q Amplitude (V)**	15.42±2.84	5.30±8.18	8.37±10.87	-100.42±78.82	-12.67±23.01	7.72±11.28
**R Amplitude (V)**	496.76±42.21	554.03±36.20	297.97±46.90$^{\#}$	371.15±46.02	492.74±49.77	450.80±52.90
**S Amplitude (V)**	-61.00±47.21	-551.7875±73.68*	-288.91±106.36	-259.20±60.37	-52.69±95.22*	-293.04±83.45
**T Amplitude (V)**	228.85±46.46	398.49±36.33$^{\%\&@}$	133.10±30.08	224.95±41.70	195.89±50.26	113.50±62.78

Supplemental Table 5: Electrocardiogram measures at 12 months of age. *p<0.05 compared to WT; $^{\#}$p<0.05 compared to untreated D2.*mdx*; $^{\%}$p<0.05 compared to MDC1; $^{\$}$p<0.05 compared to MDC2; $^{\&}$p<0.05 compared to MDC3; $^{@}$p<0.05 compared to MDC4.

	D2.WT	D2.*mdx*	MDC2	MDC3
**RR Interval (ms)**	140.91±4.06	138.92±7.60	144.20±5.91	142.63±3.41
**Heart Rate (BPM)**	428.46±11.58	441.81±22.76	420.06±16.03	422.28±9.92
**PR Interval (ms)**	37.03±3.37	34.89±0.98	35.53±1.57	34.13±1.67
**P Duration (ms)**	8.27±0.32	8.86±0.58	7.91±1.00	6.23±0.69$^{\#}$
**QRS Interval (ms)**	10.29±0.52	10.52±0.98	11.98±0.62	15.68±3.03*$^{\#}$
**QT Interval (ms)**	19.11±0.69	23.18±1.88	25.61±1.73*	19.74±0.99
**QTc (ms)**	50.95±1.70	62.21±4.41	67.81±5.09*$^{\&}$	52.10±2.75
**JT Interval (ms)**	8.82±0.51	12.67±1.48	13.63±1.23$^{\&}$	6.64±3.14
**T_{peak} T_{end} Interval (ms)**	5.78±0.42	9.32±1.45	10.09±1.02	4.75±2.09
**P Amplitude (V)**	99.03±10.19	117.94±15.84	106.68±8.32	31.67±12.08*$^{\#\$}$
**Q Amplitude (V)**	20.98±4.67	2.63±8.47	8.27±5.09	-201.49±111.41*$^{\#\$}$
**R Amplitude (V)**	492.38±40.47	418.07±54.15	326.87±46.32	640.82±91.55$^{\$}$
**S Amplitude (V)**	-121.10±93.52	-202.47±82.74	-224.53±64.17	-237.74±80.22
**T Amplitude (V)**	241.48±28.50	202.77±29.30	165.76±33.13	294.21±90.80

Supplemental Table 6: Electrocardiogram measures at 18 months of age. *p<0.05 compared to WT; $^{\#}$p<0.05 compared to untreated D2.*mdx*; $^{\$}$p<0.05 compared to MDC2; $^{\&}$p<0.05 compared to MDC3.

SUPPLEMENTAL FIGURES

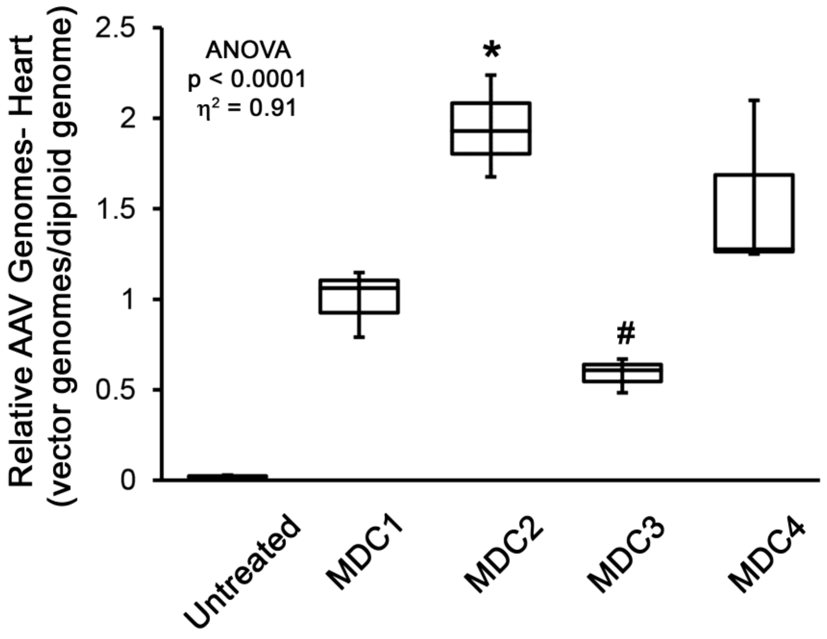

Supplemental Figure 1. AAV vector genomes were quantified in the hearts of untreated D2.*mdx* mice and those treated with $2x10^{14}$ gc/kg of MDC1, MDC2, MDC3, or MDC4 at 1 month of age using real-time PCR (n = 3 for each group). Vector genome content was normalized to murine diploid genomes using the ΔΔCT method and reported relative to MDC1 values. Data were analyzed using one-way ANOVA with Tukey HSD post-hoc tests ($\alpha = 0.05$; effect size reported at η^2; *$p < 0.05$ vs. MDC1 values; $^{\#}p < 0.05$ vs. MDC2 values) and displayed as box-and-whisker plots (boxes indicate 2nd and 3rd quartiles and error bars represent the minimum and maximum values).

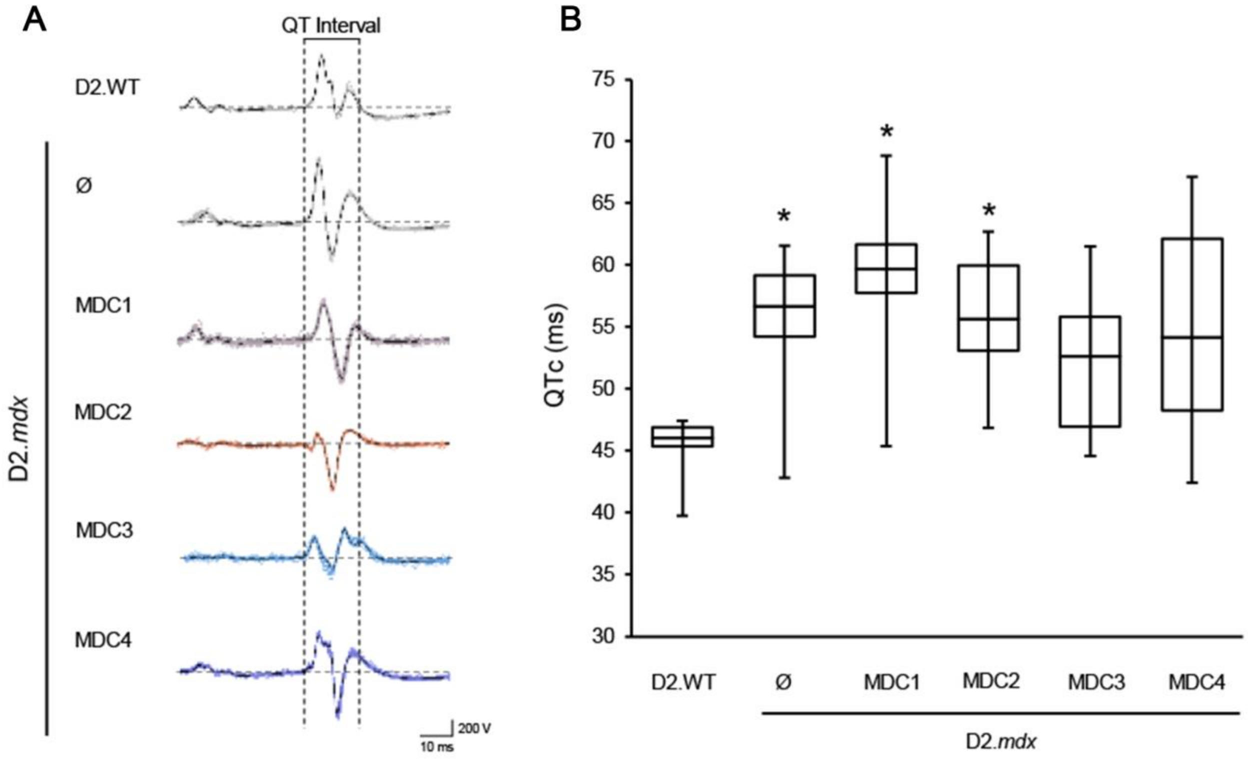

Supplemental Figure 2. Electrocardiogram abnormalities persist with micro-dystrophin treatment. Male D2.*mdx* mice were treated with micro-dystrophin (μDys) gene therapy at 1 month of age (mo; refer to **Figure 2A**). (**A**) Representative electrocardiogram tracings from 12-month-old mice reveal prolonged QT intervals in D2.*mdx* mice, even when treated with micro-dystrophin, along with (**B**) QT corrected for heart rate (QTc) quantifications to the right. *$p < 0.05$ vs. age-matched D2.WT values.

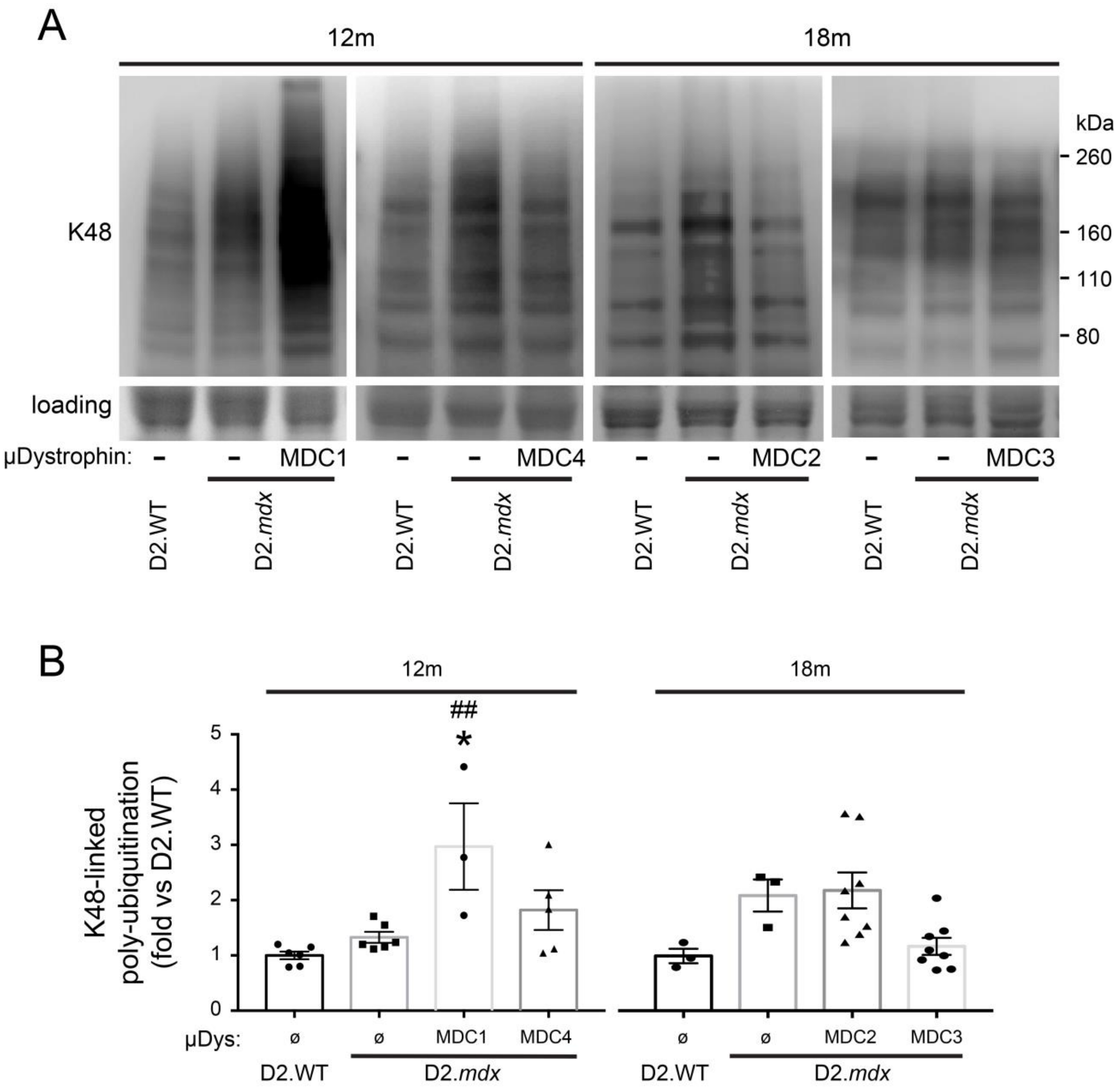

Supplemental Figure 3. Impaired protein homeostasis may further contribute to cardiac dysfunction in micro-dystrophin-treated D2.*mdx* hearts. The 5-repeat MDCs examined (MDC2-4) show similar expression in both the muscle, while the 4-repeat MDC1 was expressed at levels several-fold higher in comparison (**Fig 2B**). (**A**) End-point samples of D2.*mdx* hearts treated with micro-dystrophins – MDC1 & MDC4 at 12m and MDC2 & MDC3 at 18m – were analyzed for levels of K48-linked polyubiquitinated proteins compared to hearts of age-matched D2.WT and untreated D2.*mdx* mice. (**B**) In hearts of animals treated with micro-dystrophins MDC2 (18m; n=8), MDC3 (12m; n=5) and MDC4 (18m; n=8), no build-up of proteins destined for proteosome-mediated breakdown – evidenced by level of proteins bearing K48-linked polyubiquitin was observed above the levels observed in age matched, untreated D2.*mdx* hearts. A significant (~3-fold) increase in the build-up proteins is observed in lysates of hearts expressing MDC1 (n=3; $p<0.01$, one-way ANOVA; ## $p<0.01$ vs D2.WT, * $p<0.05$ vs D2.*mdx*, Tukey post-hoc comparison).

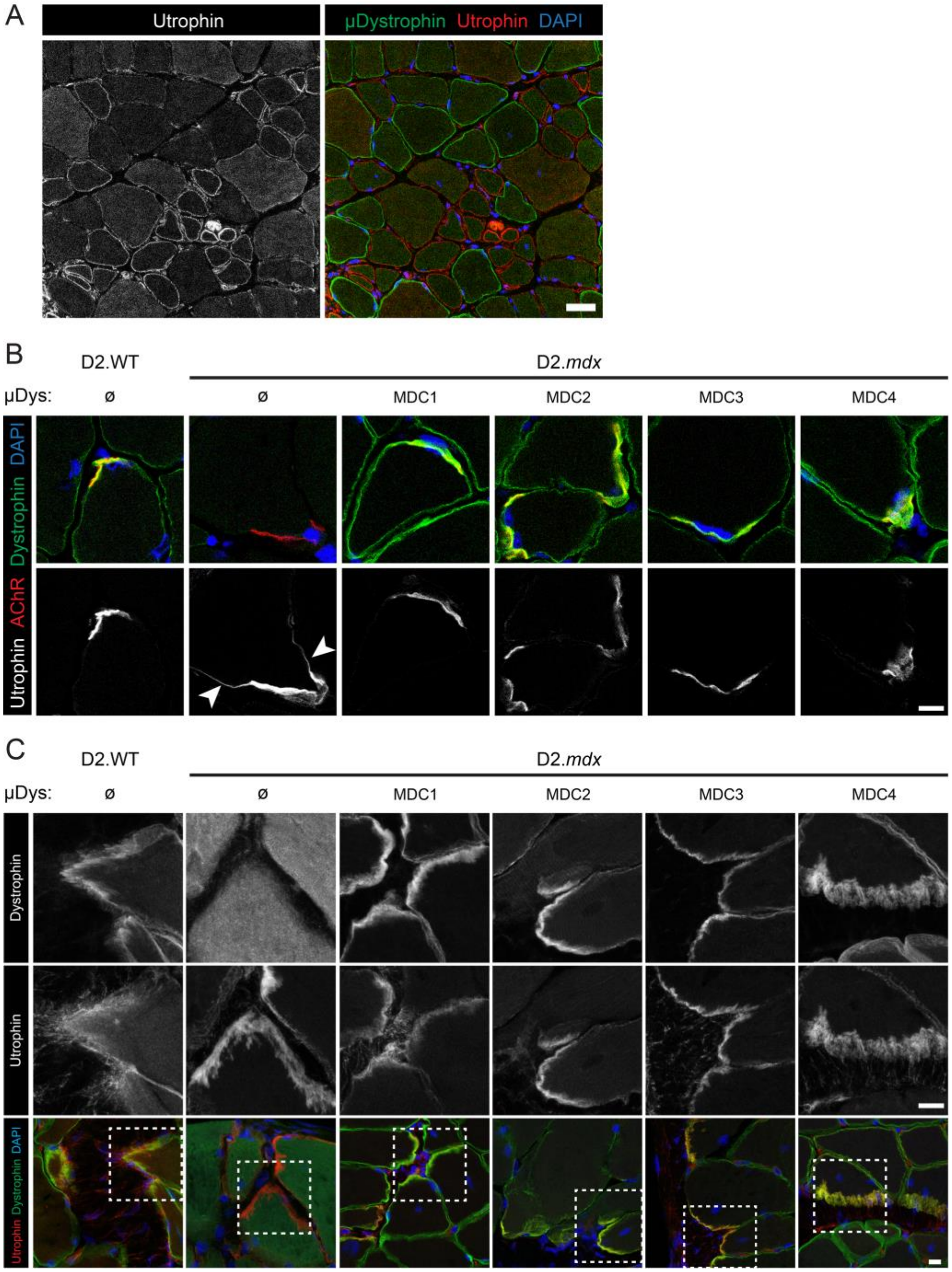

Supplemental Figure 4. Micro-dystrophin overexpression in skeletal muscle fibers displaces utrophin from sarcolemma outside of neuromuscular and myotendinous junctions. (**A**) Micro-dystrophin and utrophin demonstrate a mutually exclusive pattern of sarcolemmal labeling amongst skeletal muscle fibers in mice treated with MDC4. (B,C) Utrophin distribution is restricted to neuromuscular junctions (NMJs) (**B**) and (**C**) myotendinous junctions (MTJs) in myofibers of D2.WT mice. Utrophin labeling extends beyond the postsynaptic membrane (white arrowhead) in D2.*mdx* skeletal muscle fibers. Utrophin remains concentrated at NMJs (as indicated by co-localization with postsynaptic acetylcholine receptors (AChR, red) or MTJs of muscle fibers overexpressing any of the micro-dystrophins examined. Scale bars: 10 μm (**A-B**); 20 μm (**C**)

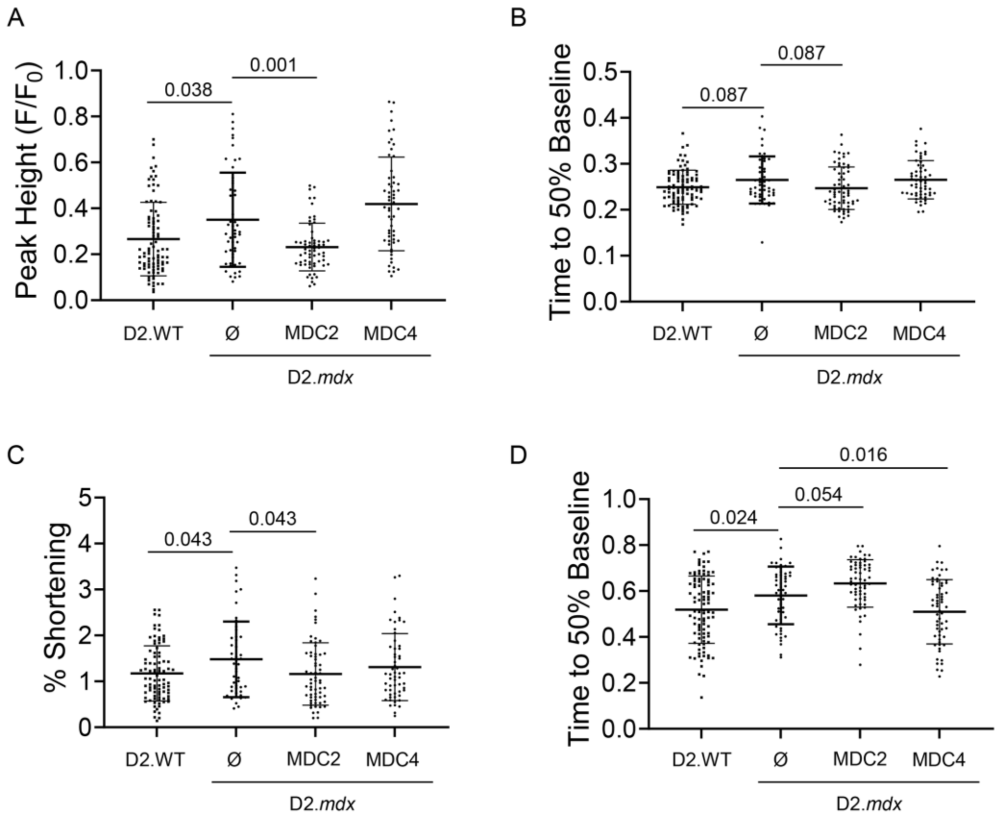

Supplemental Figure 5. MDC2 and MDC4 exert differential impacts on Ca^{2+} transients and contractility in dystrophic cardiomyocytes. Calcium transients were assessed in 12-month-old D2.WT, D2.*mdx*, and MDC2 and MDC4 treated D2.*mdx* mice. (**A**) MDC2 normalized peak Ca^{2+} release and MDC4 increased peak Ca^{2+} release. (**B**) Ca^{2+} reuptake kinetics were not significantly different between groups (**C**) MDC4 normalized %shortening in dystrophic cardiomyocytes. (**D**) MDC4 normalized relaxation kinetics and MDC2 increased relaxation kinetics. One-way ANOVA, Holm-Sidak multiple comparisons vs D2.*mdx* (**B, C**) and Brown-Forsythe and Welch ANOVA vs. D2.*mdx* (**A, D**).
