# Supplementary material for: Potential limitations of microdystrophin gene therapy for Duchenne muscular dystrophy
Source: JCI Insight. 2024 May 7;9(11):e165869. doi: 10.1172/jci.insight.165869 (PMC11382885; doi:10.1172/jci.insight.165869)
Supplement: Unedited blot and gel images [file jciinsight-9-165869-s129.pdf]

Figure 2C – heart

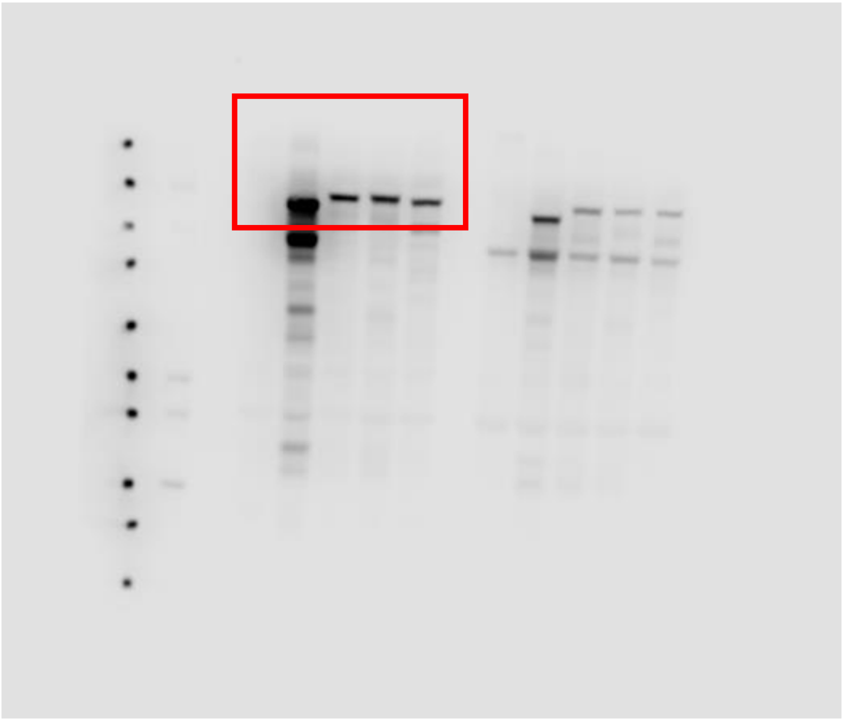

Mixture of: (Developmental Studies Hybridoma bank)

MANHINGE1B (Clone 10F9)
MANEX1011B (Clone 1C7)
MANEX1011C (Clone 4F9)

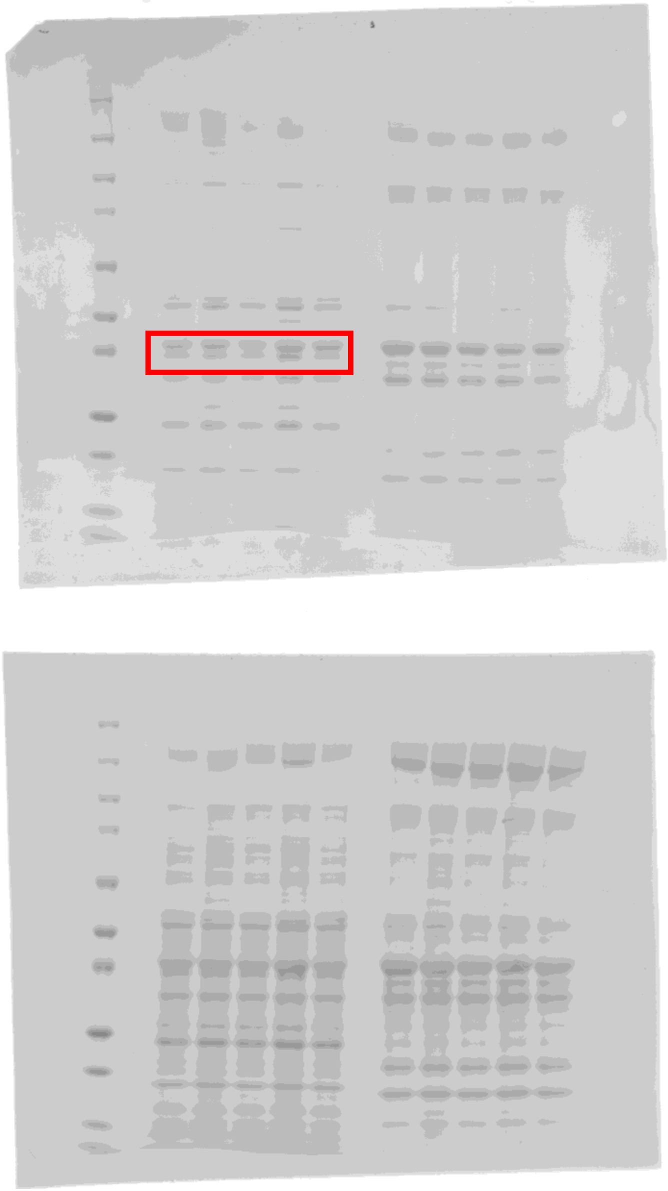

Ponceau S

Figure 2C – gastrocnemius

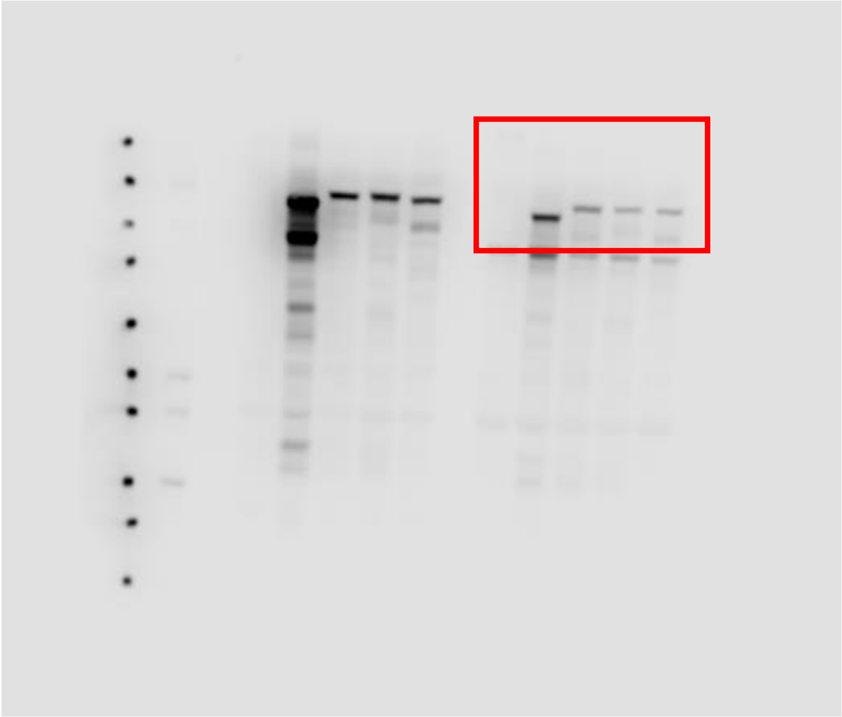

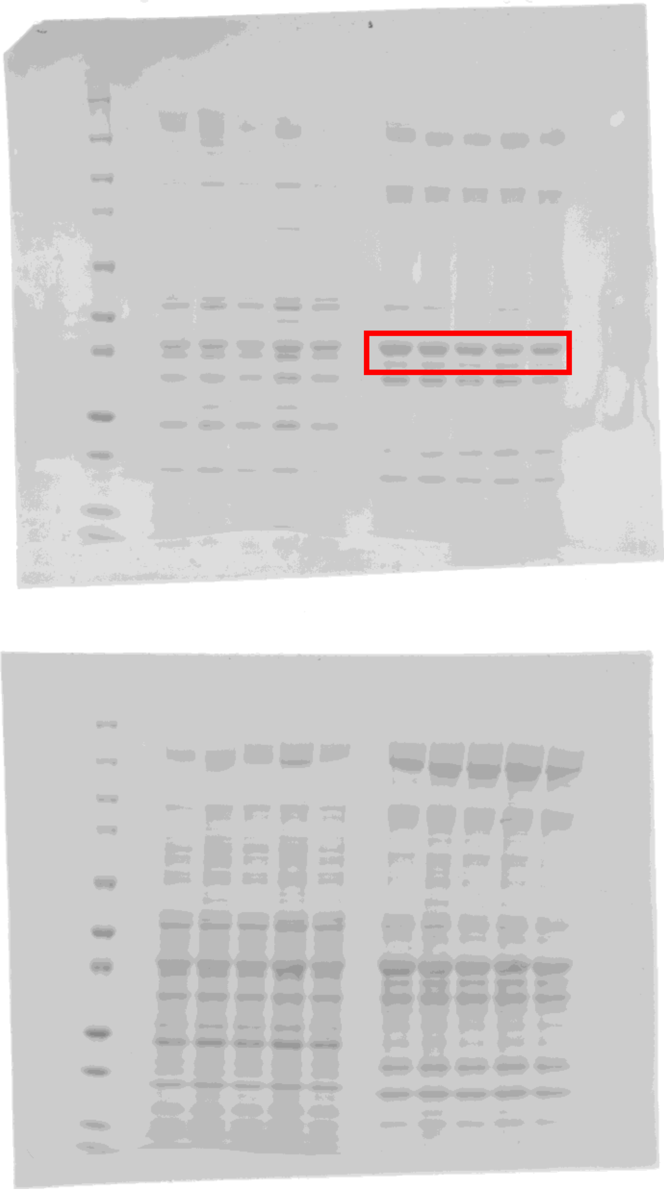

Mixture of: (Developmental Studies Hybridoma bank)

MANHINGE1B (Clone 10F9)
MANEX1011B (Clone 1C7)
MANEX1011C (Clone 4F9)

Ponceau S

Figure 2D – heart

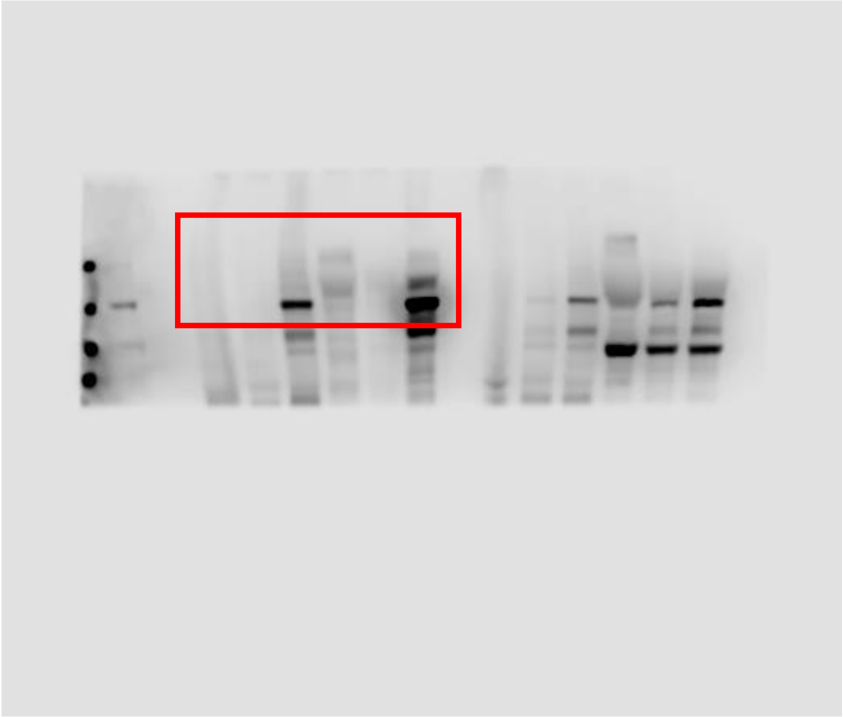

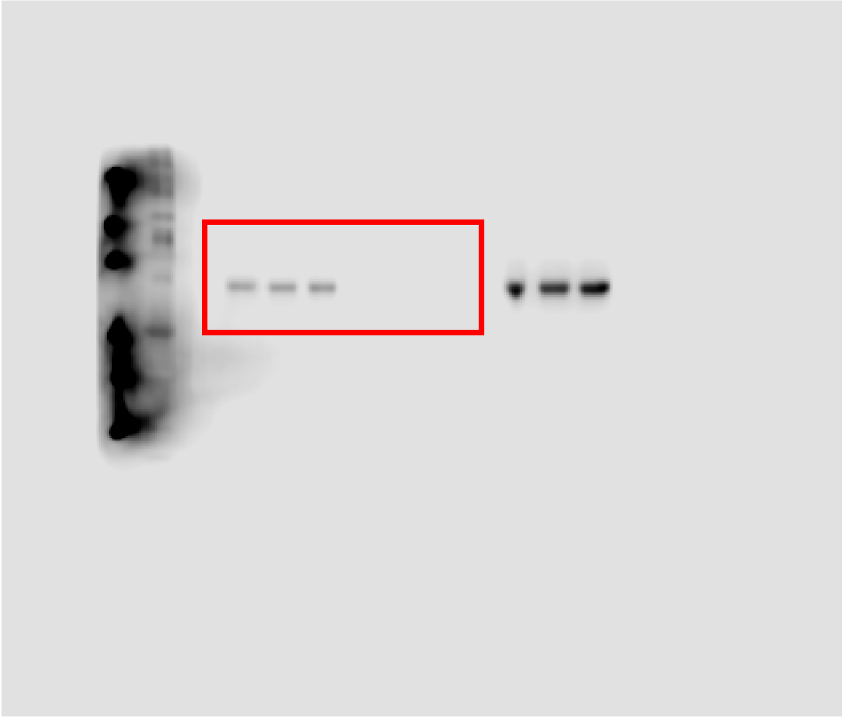

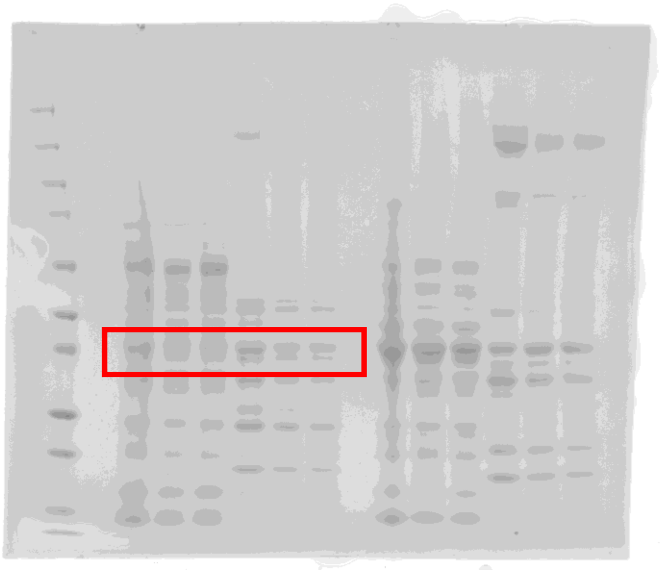

Ponceau S

Mixture of: (Developmental Studies Hybridoma bank)

MANHINGE1B (Clone 10F9)
MANEX1011B (Clone 1C7)
MANEX1011C (Clone 4F9)

GAPDH (SC-25778; Santa Cruz BioTech)

Figure 2D – gastrocnemius

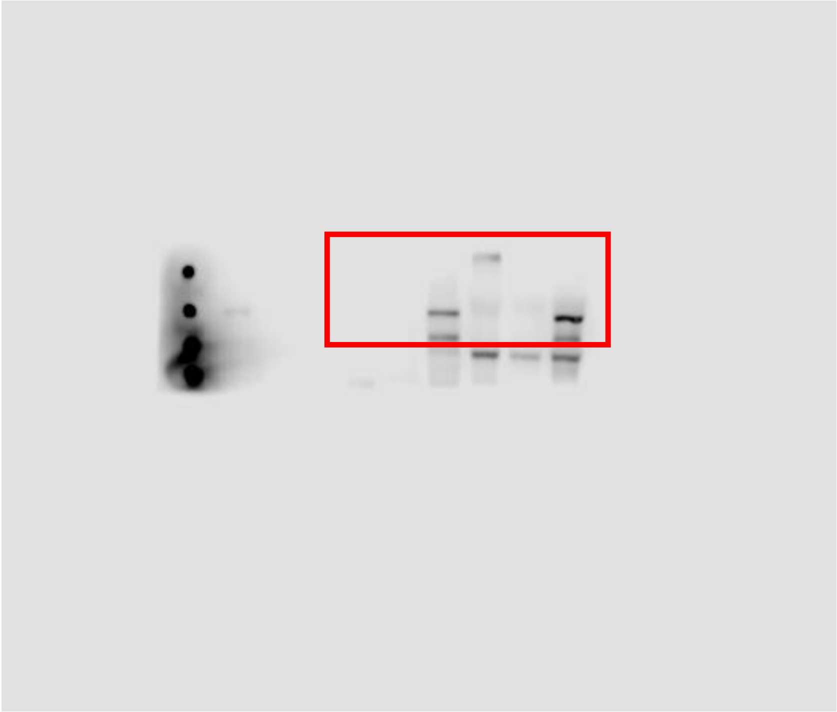

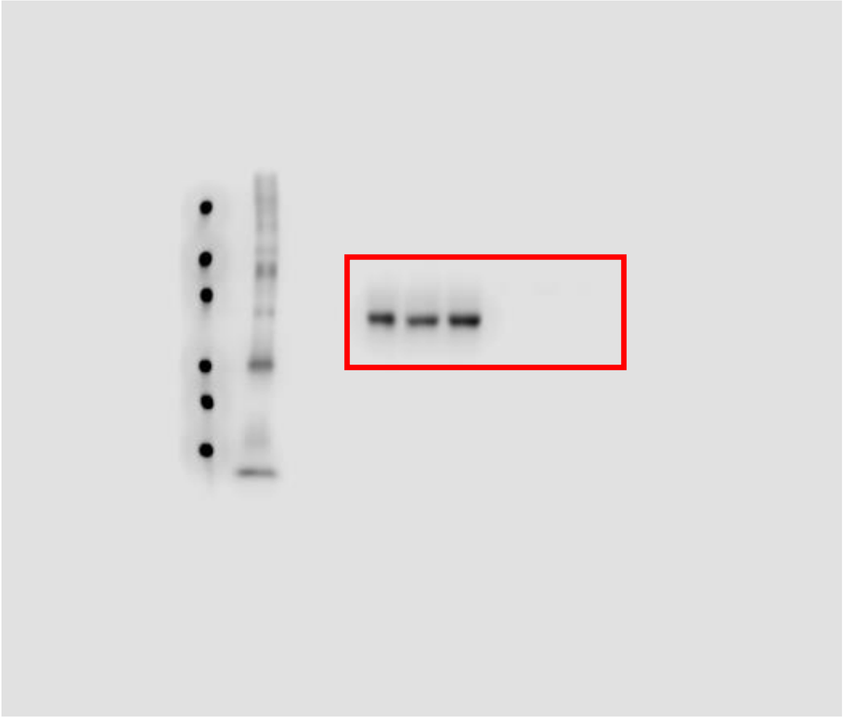

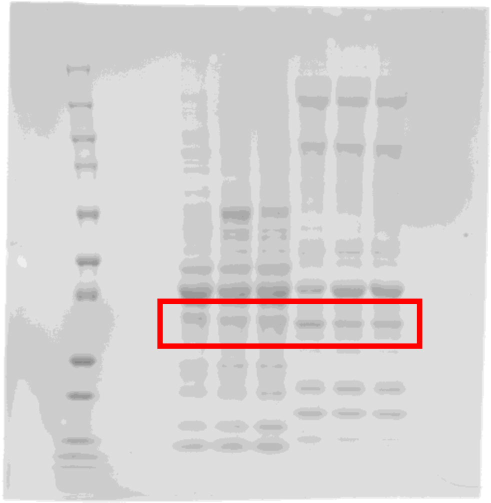

Ponceau S

Mixture of: (Developmental Studies Hybridoma bank)

MANHINGE1B (Clone 10F9)
MANEX1011B (Clone 1C7)
MANEX1011C (Clone 4F9)

GAPDH (SC-25778; Santa Cruz BioTech)

Figure 5A – MDC1

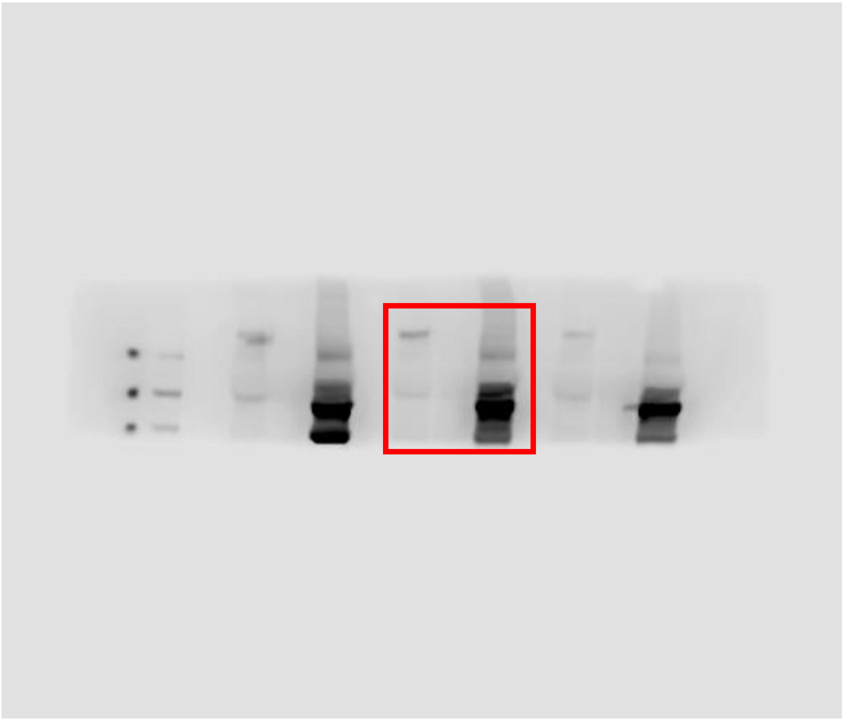

Mixture of: (Developmental Studies Hybridoma bank)

MANHINGE1B (Clone 10F9)
MANEX1011B (Clone 1C7)
MANEX1011C (Clone 4F9)

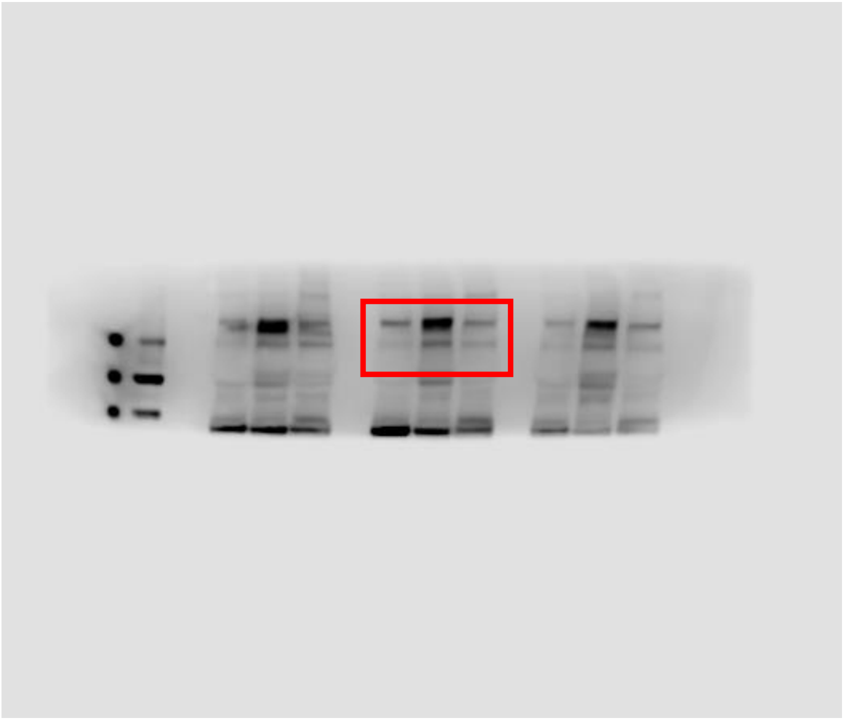

Utrophin-A (ABN1739; EMD Millipore)

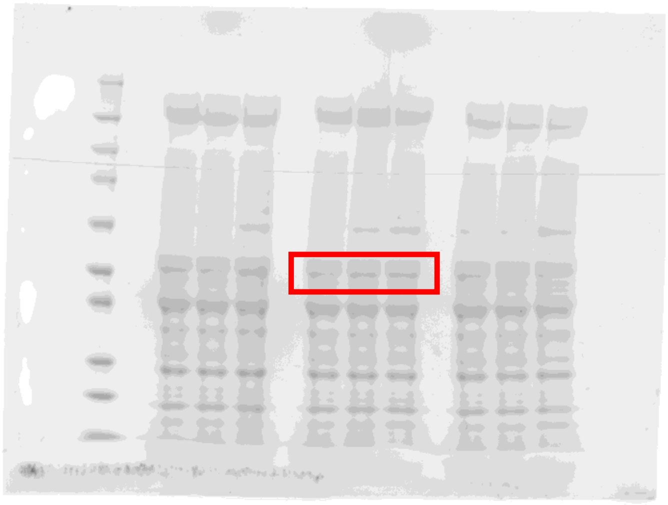

Ponceau S

Figure 5A – MDC4

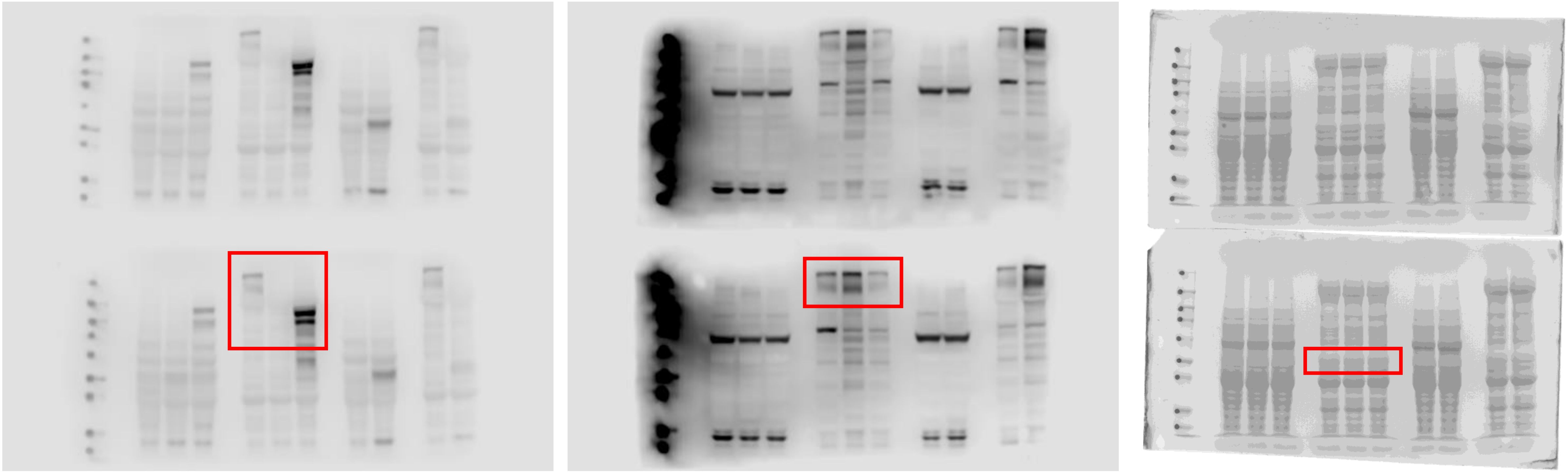

Mixture of: (Developmental Studies Hybridoma bank)

MANHINGE1B (Clone 10F9)
MANEX1011B (Clone 1C7)
MANEX1011C (Clone 4F9)

Utrophin-A (ABN1739; EMD Millipore)

Ponceau S

Figure 5A – MDC2

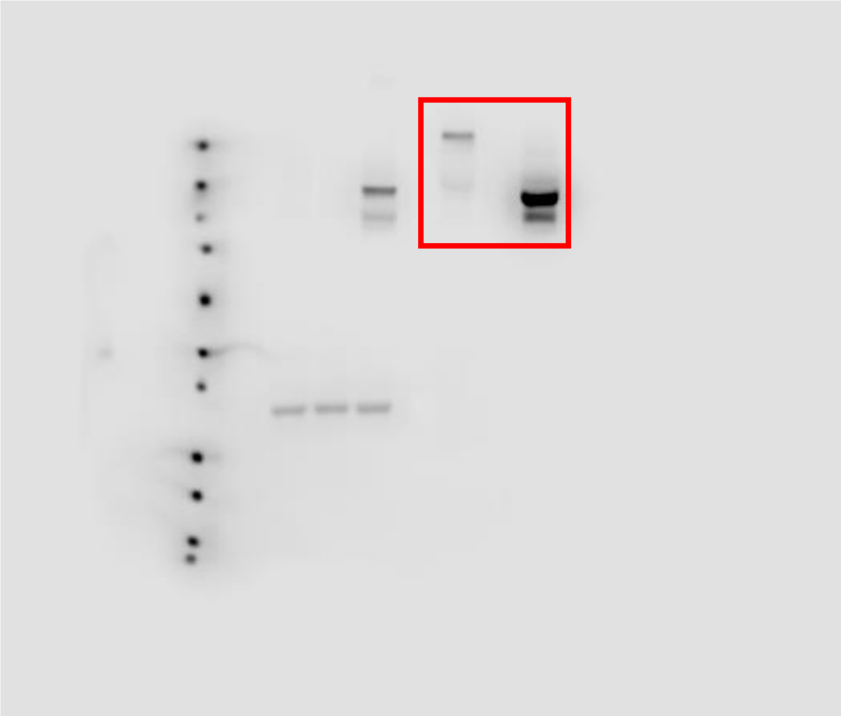

Mixture of: (Developmental Studies Hybridoma bank)

MANHINGE1B (Clone 10F9)
MANEX1011B (Clone 1C7)
MANEX1011C (Clone 4F9)

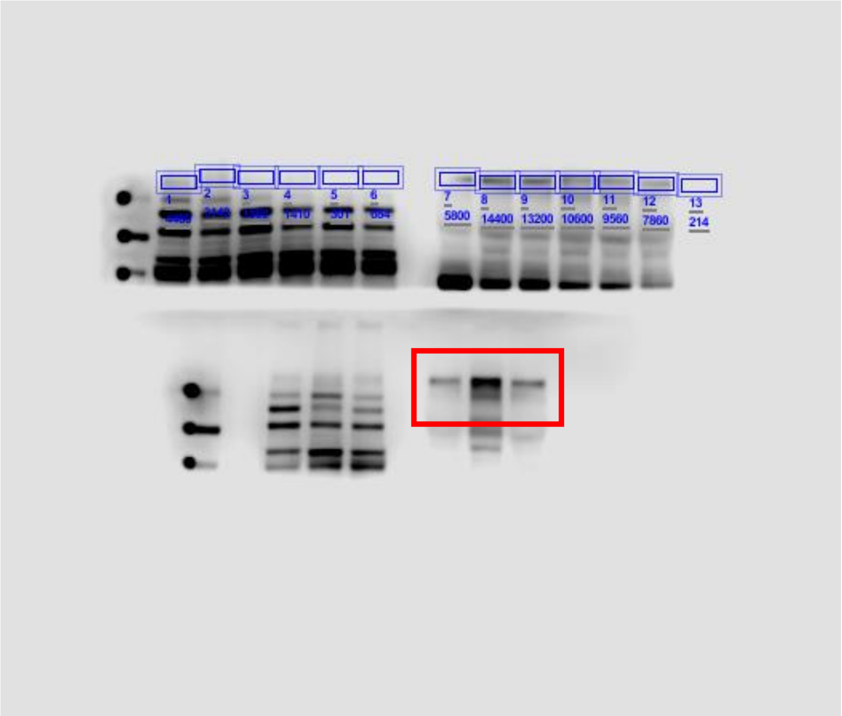

Utrophin-A (ABN1739; EMD Millipore)

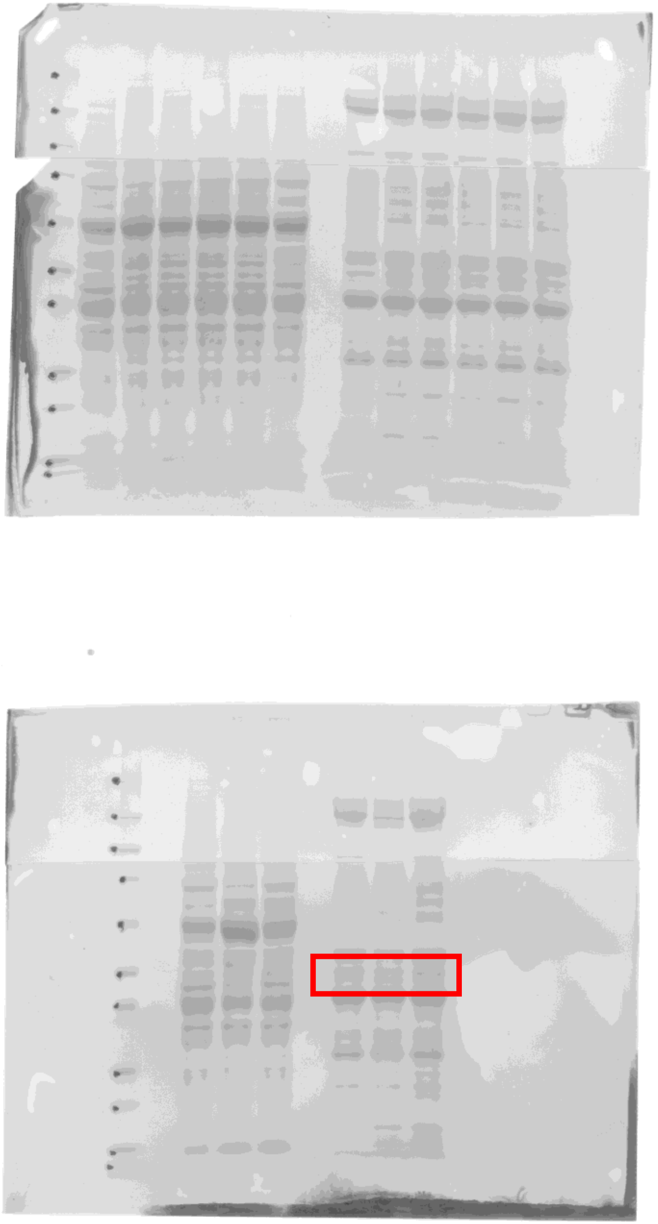

Ponceau S

Figure 5A – MDC3

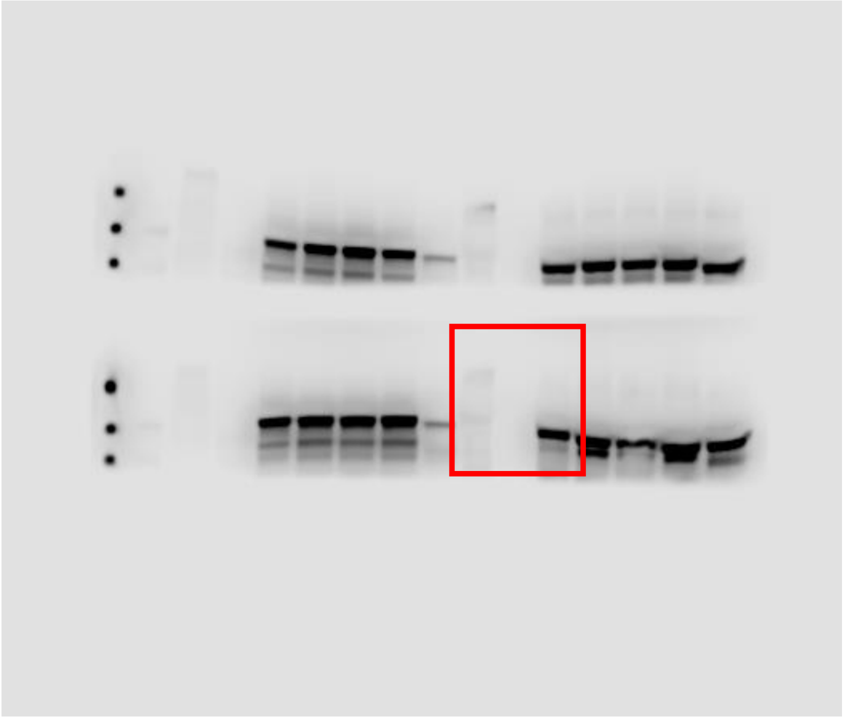

Mixture of: (Developmental Studies Hybridoma bank)

MANHINGE1B (Clone 10F9)
MANEX1011B (Clone 1C7)
MANEX1011C (Clone 4F9)

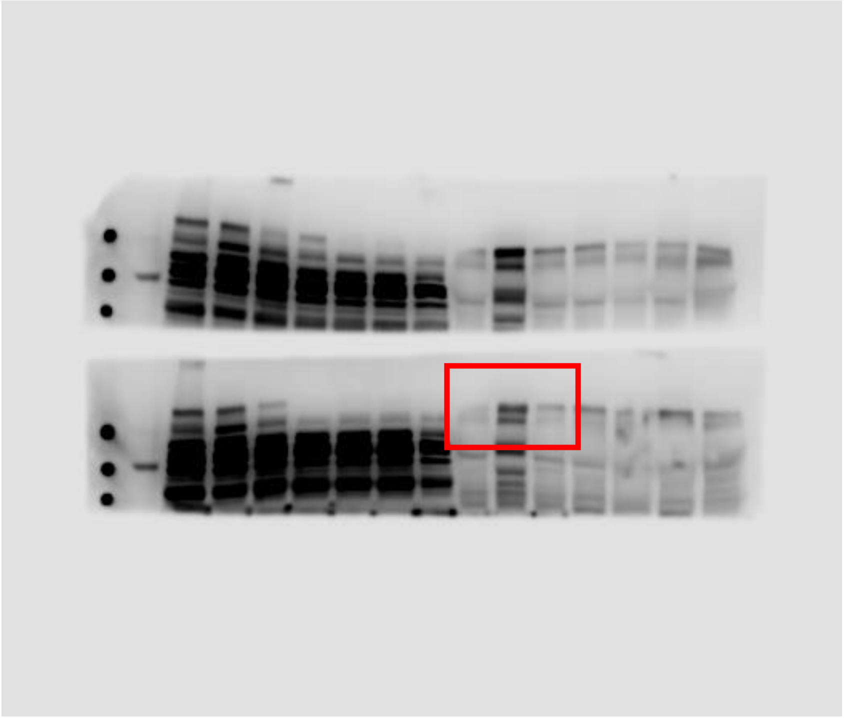

Utrophin-A (ABN1739; EMD Millipore)

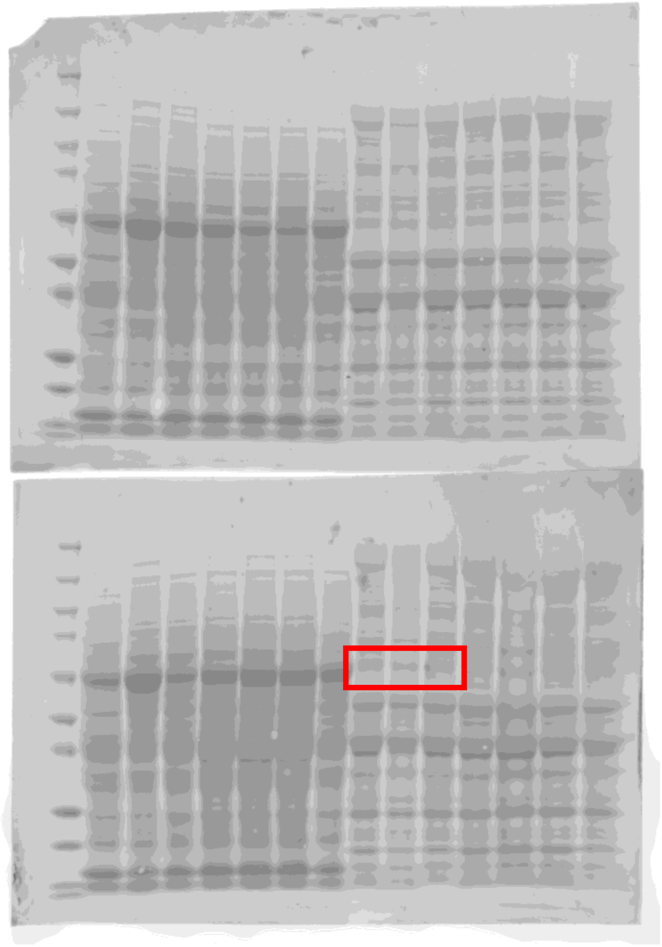

Ponceau S

Supplemental Figure 2A – MDC1

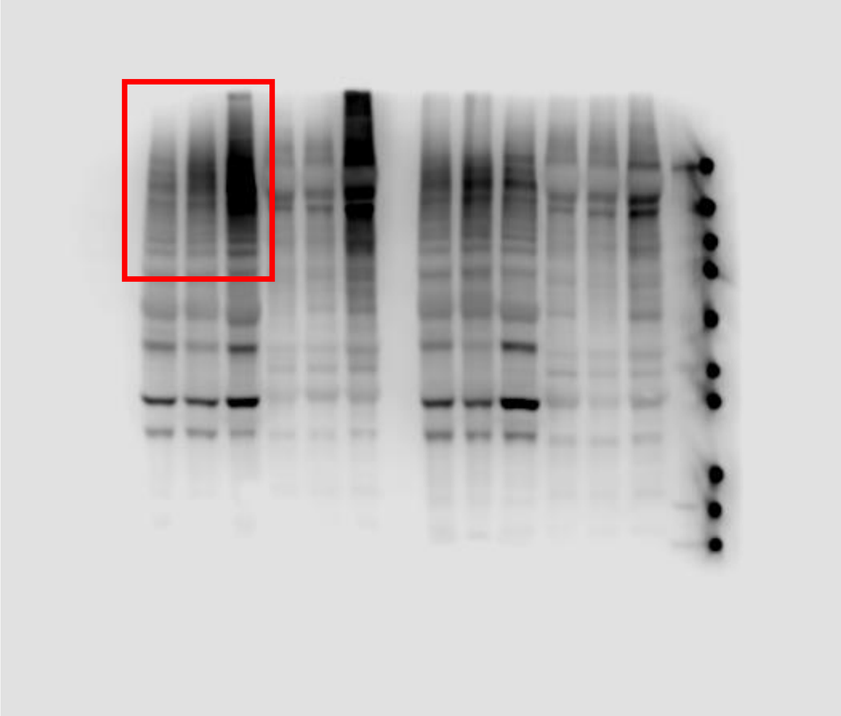

Polyubiquitin (K48-linkage; #4389, Cell Signaling)

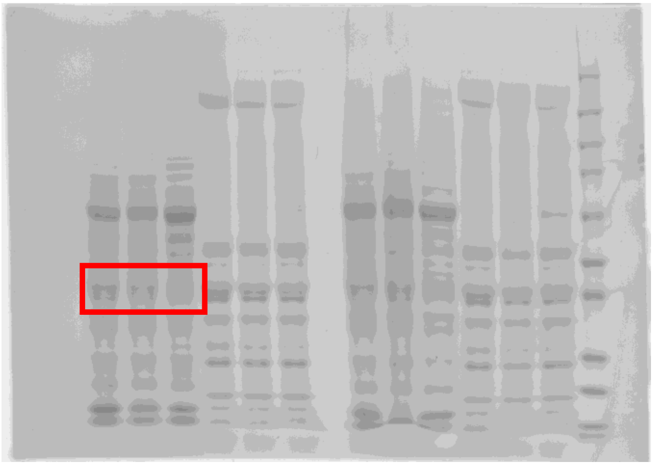

Ponceau S

Supplemental Figure 2A – MDC4

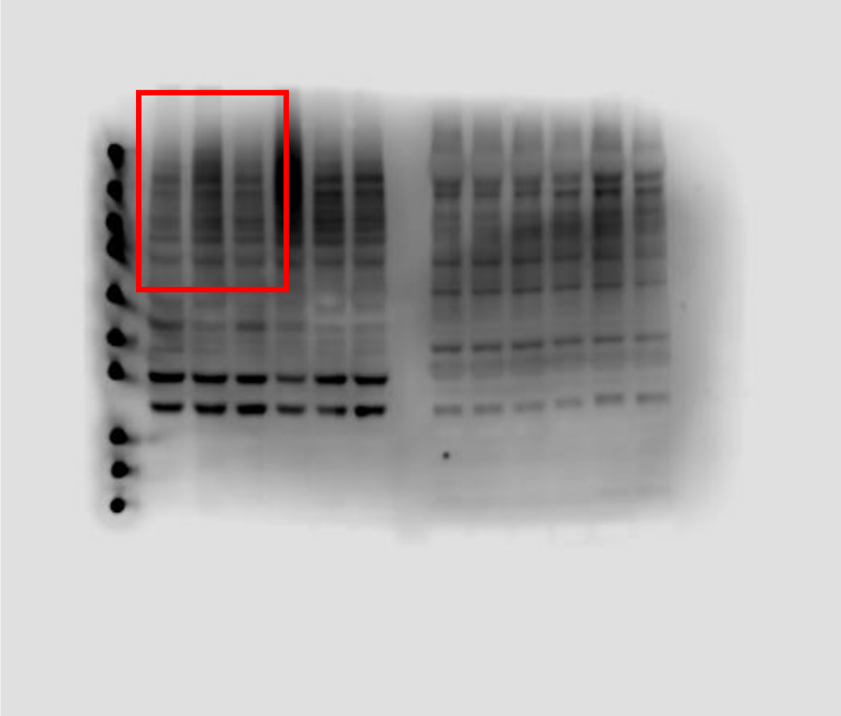

Polyubiquitin (K48-linkage; #4389, Cell Signaling)

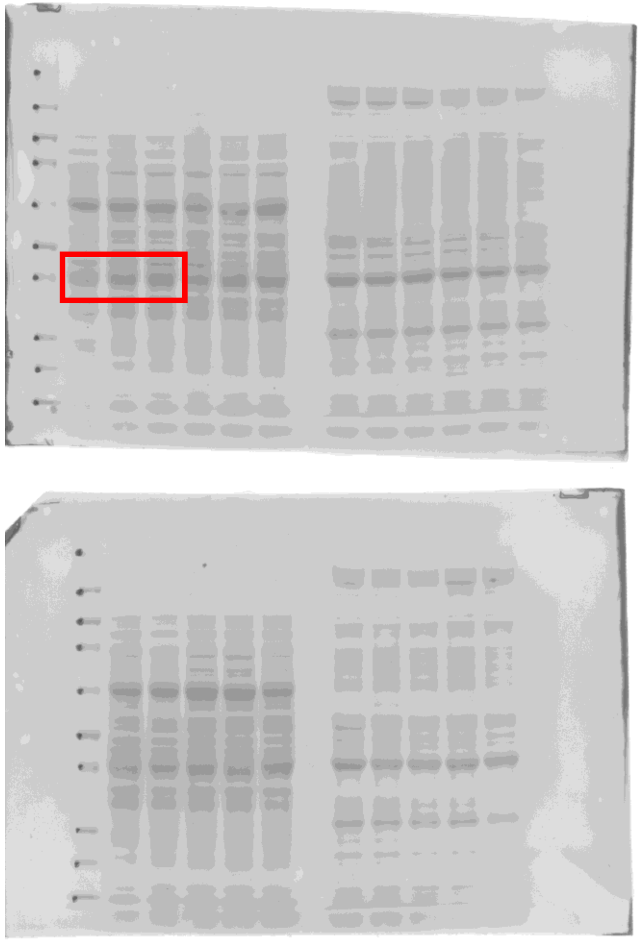

Ponceau S

Supplemental Figure 2A – MDC2

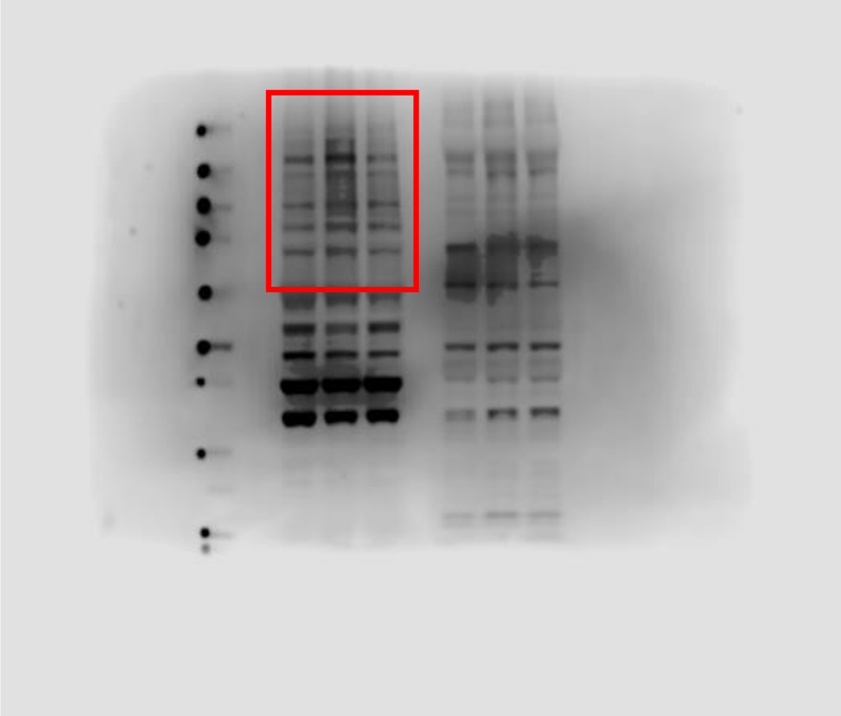

Polyubiquitin (K48-linkage; #4389, Cell Signaling)

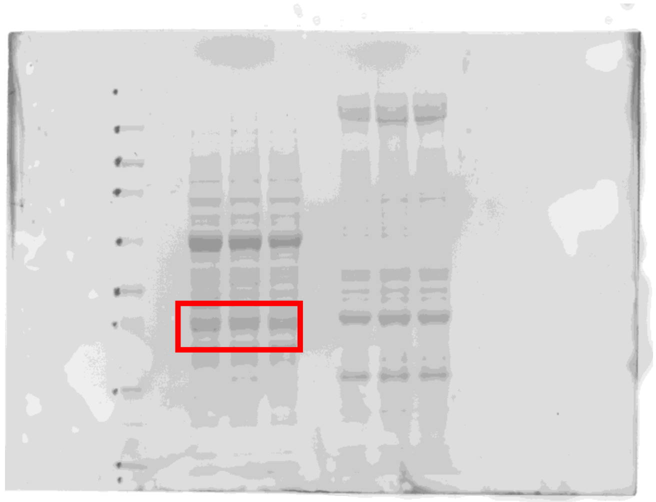

Ponceau S

Supplemental Figure 2A – MDC3

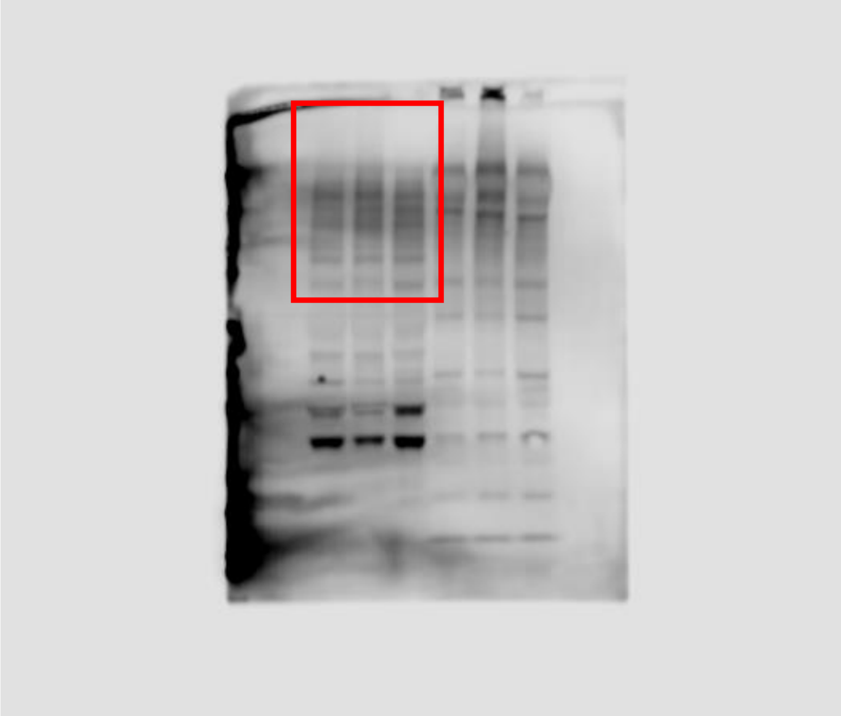

Polyubiquitin (K48-linkage; #4389, Cell Signaling)

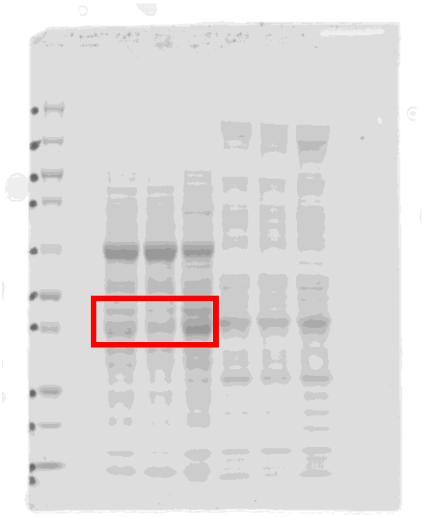

Ponceau S
